# Methodologies and methods for the development, evaluation and implementation of psychosocial interventions for dementia: protocol for a scoping review

**DOI:** 10.1136/bmjopen-2025-114584

**Published:** 2026-04-24

**Authors:** Federica D’Andrea, Sara Laureen Bartels, Marine Markaryan, Patricia Masterson-Algar, Andrea Nakakawa Bernal, Simone R De Bruin, Ilaria Chirico, Aisling Flynn, Lesley Garcia, Doris Gebhard, Melanie Handley, Niels Janssen, Martina Roes, Nathan Stephens, Gwen Teesing, Lieve Van den Block, Karen Windle, Esme Moniz-Cook, Maud Graff

**Affiliations:** 1 The Geller Institute of Ageing and Memory, School of Medicine and Biosciences, University of West London, London, UK; 2 Department of Psychiatry and Neuropsychology and Alzheimer Centrum Limburg, Mental Health and Neuroscience Research Institute, Maastricht University, Maastricht, The Netherlands; 3 Department of Clinical Neuroscience, Karolinska Institutet, Stockholm, Sweden; 4 School of Health Sciences, Bangor University, Bangor, UK; 5 Department of Design, Politecnico di Milano, Milan, Italy; 6 Department of Health and Social Care, Research Group Living Well with Dementia, Windesheim University of Applied Sciences, Zwolle, The Netherlands; 7 Department of Human Sciences for Education ‘Riccardo Massa’, University of Milano, Bicocca, Italy; 8 Department of Rehabilitation and Sports Science, Bournemouth University, Poole, UK; 9 School of Sociology and Social Policy, University of Nottingham, Nottingham, UK; 10 Department of Health and Sport Sciences, Technical University of Munich, Munchen, Germany; 11 Centre for Research in Public Health and Community Care, University of Hertfordshire, Hatfield, UK; 12 Witten Site, German Centre for Neurodegenerative Diseases, Bonn, Germany; 13 Department of Nursing Science, University Witten Herdecke Faculty of Health, Witten, Germany; 14 The Association for Dementia Studies, University of Worcester, Worcester, UK; 15 School of Social Policy, Improving Adult Care Together, University of Birmingham, Birmingham, UK; 16 Alzheimer Nederland, Amersfoort, The Netherlands; 17 Department of Family Medicine and Chronic Care, VUB-UGent End-of-Life Care Research Group, Vrije Universiteit Brussel, Brussels, Belgium; 18 Faculty of Life and Health Sciences, University of Ulster, Coleraine, UK; 19 Faculty of Health Sciences, University of Hull, Hull, UK; 20 Department of Rehabilitation, Radboud University Medical Center, Nijmegen, The Netherlands; 21 Department of IQ Health, Radboud University Medical Center, Nijmegen, The Netherlands; 22 Radboudumc Alzheimer Center, Radboud University Medical Center, Nijmegen, The Netherlands

**Keywords:** Dementia, Psychosocial Intervention, Methods, Feasibility Studies, Implementation Science, Review

## Abstract

**Abstract:**

**Introduction:**

Research on psychosocial interventions for dementia demonstrates increased rigour and robustness. However, if we are to influence practice, beyond results from randomised controlled trials, a variety of types and sources of evidence is needed. The Medical Research Council (MRC) framework offers a valuable guide for developing, evaluating and implementing complex interventions, to facilitate integration of research into practice. There is limited knowledge of how researchers design, evaluate and implement psychosocial intervention studies in dementia, using the MRC framework. This scoping review aims to: (1) identify the methodological and methods trends, use and gaps in the development, evaluation and implementation of psychosocial interventions for dementia, and (2) determine if and how the MRC six core elements were considered and applied in studies.

**Methods and analysis:**

Six databases (Ovid MEDLINE, Embase, PsycINFO, CINAHL, Web of Science, Cochrane Library) will be searched for studies published from 2015 (when MRC process guidance was published) to 2025. Identified deduplicated citations will be imported into Covidence software, where up to 40% of title/abstracts will be double screened by independent reviewers. ASReview will be used to rank articles by relevance, with a stopping criterion of 250 consecutive irrelevant articles. Full texts will be reviewed by a single reviewer and those excluded will be checked by a second reviewer. Data extraction will include study aim/objective (ie, to develop/adapt; test feasibility/pilot; evaluate; implement); methodology and methods applied; information on which MRC six core elements were considered (yes/no), and if so, how they were addressed (ie, qualitative details). A narrative synthesis, alongside graphical representations (eg, table/bar charts/histograms), will be used to synthesise findings on methodologies and methods mapped onto the MRC framework.

**Ethics and dissemination:**

This secondary analysis scoping review does not require ethics approval. Results will be disseminated through peer-reviewed publication(s), seminars, webinars, conferences, postgraduate dementia programmes, blogs, commissioner briefings and social media. The findings will provide a state-of-the-art overview of current practices; advance methods/methodology such as informing a Delphi consensus study on appropriate research approaches; and guide researchers in application of the MRC framework to widen the scope of dementia care evidence for practice improvements.

**Registration:**

Submitted to Open Science Framework https://doi.org/10.17605/OSF.IO/S56NQ.

**STRENGTHS AND LIMITATIONS OF THIS STUDY**

- This review uses the updated Medical Research Council Framework which provides a rigorous structure for synthesising methodologies (eg, theoretical framework/paradigms, research approach/design) and methods (eg, data collection/analysis).
- Covidence software and the ASReview tool will be used to enhance transparency, rigour and efficiency of evidence screening.
- The review focuses on methodologies and methods used in studies of psychosocial intervention in dementia rather than their outcomes.
- Restriction of publication language to English only is a limitation of this study.
- Exclusion of studies on rarer diseases and conditions leading to dementia and/or dementia-like symptoms may limit generalisability of findings.

## Introduction

 In dementia care, psychosocial interventions are essential for promoting, supporting or sustaining cognition, everyday functioning, well-being and interpersonal relationships.^1^,^4^ Commonly, these interventions are also referred to as ‘non-pharmacological’ and encompass interactions between people, self-management activities, techniques, strategies, training or technology involving sensory, cognitive, physical, behavioural, emotional, educational, social and/or environmental components.^5 6^ Psychosocial interventions for dementia target a range of outcomes, groups, settings and organisational levels. They often require expertise and skills to deliver and allow for flexibility within the intervention or its components. Psychosocial interventions may be single-component or multicomponent, but they are always complex in nature because of the intervention characteristics (eg, number of components, behaviours targeted) as well as how these characteristics interact with the inner (core) and outer (systemic) intervention context.^7^ To generate robust and useful evidence on which psychosocial interventions for dementia work (or not), for whom, how and under which circumstances, the suitability of the methodological approach for specific research questions remains important.^8^ For example, dementia researchers may aim to develop or adapt a psychosocial intervention to a specific setting or population,^9 10^ investigate if and how delivery of an intervention can be feasible and acceptable,^11 12^ determine which effects the intervention has on selected outcomes^5^ or explore implementation success.^13^ These examples of research questions are in line with the phases of the Medical Research Council (MRC) framework for developing and evaluating complex interventions,^14^ namely (1) develop/identify intervention, (2) feasibility/piloting, (3) evaluation and (4) implementation. Box 1 provides definitions of these terms for clarity.

Box 1 **Definition of development/identification, feasibility/pilot, evaluation and implementation studies**

- Development or identification: ‘The whole process of designing and planning an intervention’ (p.6),^14^ especially the conceptualisation, including stakeholder involvement, development of theoretical frameworks and decision-making process; may focus on creating a new intervention or adapting and testing an existing intervention to a new context of target group.
- Feasibility and pilot study^48^: ‘Conducted to determine the feasibility or acceptability of an intervention or evaluation design in order to make decision about progression to the next stage of evaluation’ (p.4).^14 49^
  - Feasibility: Focuses on the question ‘Can this be done?’ and aims at exploring the possibility of carrying out an evaluation study of a psychosocial intervention in a specific context, determining if it is worth proceeding (eg, likelihood of cost effectiveness), and if so, how it should be done by estimating uncertain parameters, such as the willingness of participants to participate in the intervention (demand, acceptability), willingness to be randomised, recruitment, response, treatment delivery, adherence and compliance rates.^50 51^
  - Pilot study: Small-scale version of a planned future trial, conducted to assess whether the components of the main study are appropriate. This includes recruitment, randomisation, intervention components (such as treatment delivery and adherence), implementation aspects (strategies and requirements), outcome measures and follow-up. A pilot study might include the evaluation of intervention outcomes.^52^
- Evaluation: Study to enable judgement about the value (eg, effectiveness/efficacy/cost benefits) of the intervention; determine whether or not an intervention achieves its intended outcomes; explore if the intervention is superior or equally beneficial to another intervention; identify unanticipated adverse impacts; theorise how the intervention impacts outcomes; explore how it interacts with the context in which it is implemented; determine if it contributes to system changes; or decide how the evaluation evidence can be used to support real-world decision making.^14^
- Implementation: ‘Deliberate effort to increase the impact and uptake of successfully tested health innovations’, (p.5) with the goal of benefitting larger groups and promoting sustainable policy and programme development,^53^ with key outcomes including acceptability, adoption, appropriateness, feasibility, fidelity, implementation costs, penetration and sustainability.^54^,^57^

**Figure 1:**
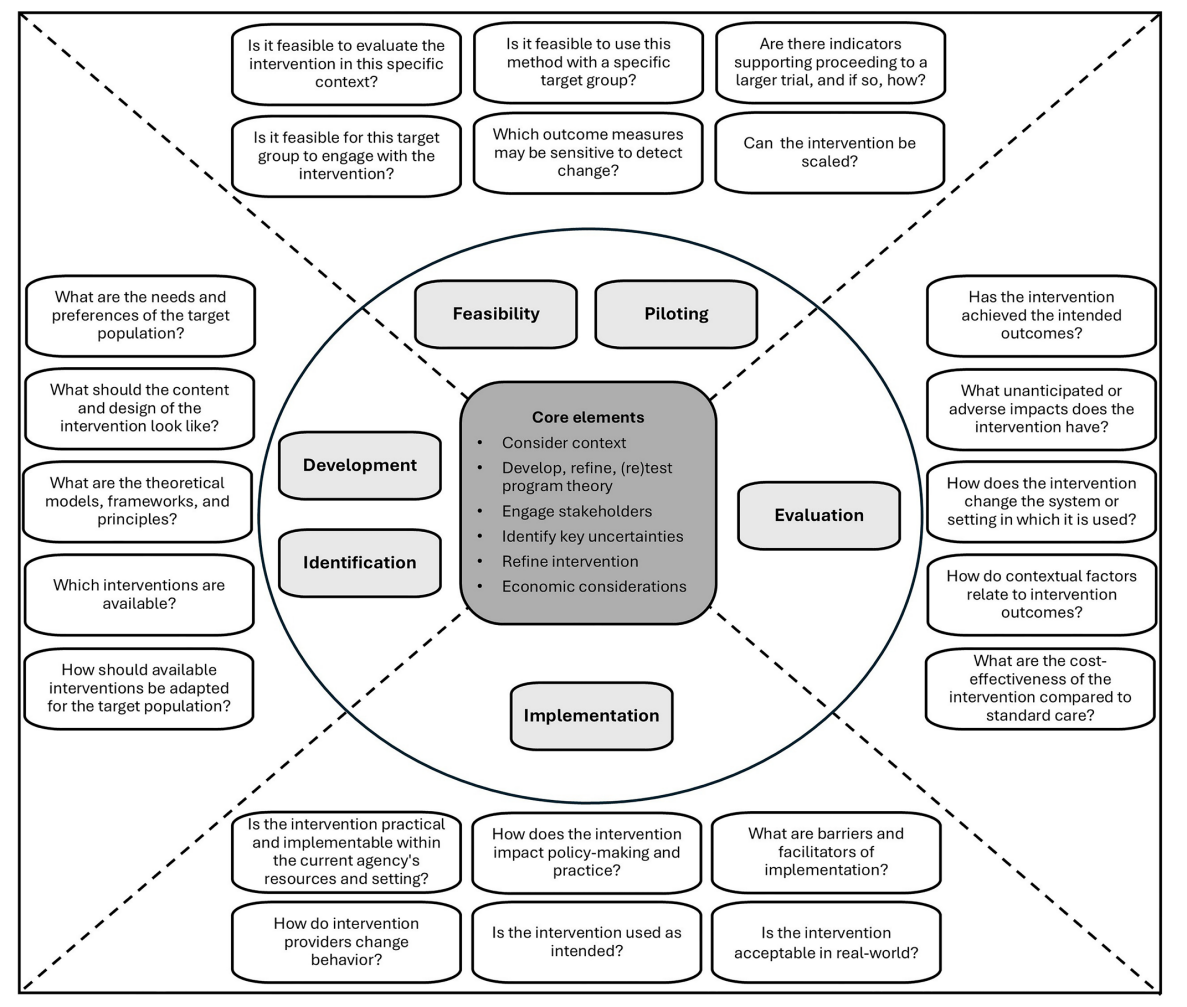
Visualisation of the core elements and phases for research on psychosocial interventions for dementia, adapted from the MRC framework ^14^ (CC BY 4.0 licence), including examples of research questions. MRC, Medical Research Council.

These four phases are non-linear and can also be combined, eg, during a process evaluation. Moreover, six core elements are emphasised as being central processes across the four phases: (1) consider context, (2) develop, refine and (re)test programme theory, (3) engage stakeholders, (4) identify key uncertainties, (5) refine intervention and (6) economic considerations.^7 14^ Figure 1 provides an overview of the MRC framework^14^ adapted by the authors including examples of research questions per phase.

Recently, methodological gaps in psychosocial intervention research have been highlighted, including (1) the lack of meaningful involvement of people with dementia, unpaid carers (such as family members, relatives, partners/significant others, friends or neighbours) and other stakeholders, (2) neglect of context and intervention complexity, and (3) methods not detecting or capturing what works (or not), for whom, how and why.^8^ These gaps experienced by dementia researchers, and by people with dementia, unpaid carers and other stakeholders, could be linked to the over-reliance of policymakers and external funders on Randomised Controlled Trials (RCTs) (RCTs assess intervention efficacy within ‘ideal world’ or optimum conditions.^15^ Consequently, there is a risk of research waste due to an ‘implementation error’ where (1) costly evaluations may fail to demonstrate effectiveness despite positive impacts on peoples’ experiences or (2) may prove effective yet remain unfeasible in real-world practice^16^.)^17^ Generating complementary knowledge to inform the field beyond traditional effectiveness trials (eg, RCTs) is important in developing the evidence for relevant psychosocial interventions that are acceptable to people with dementia and families and that can be implemented at scale in real-world settings. This may additionally reduce research waste and improve care across the dementia trajectory.^8^ Other fields have reflected and discussed how the MRC framework can be applied, resulting in clear suggestions for future studies (eg, studies by Skivington *et al*^7^ and Pinto *et al*^18^). To date, there is little guidance for dementia researchers on how MRC core elements might be addressed within predefined project timelines and funding constraints. The global burden of dementia remains significant, and the role of age-related multimorbidity is not often clear.^19 20^ In the absence of a cure, a comprehensive overview exploring how the MRC framework is applied to psychosocial dementia studies may have scope to widen the debate surrounding evidence-based health and social care associated with dementia and potentially other areas of ill health.^8^

### The overarching objective of the present scoping review

The MRC framework is one of the most referenced frameworks, and its core elements appear highly relevant for developing, evaluating and implementing complex psychosocial interventions for dementia. It was conceived some 25 years ago and its refinement over time reflects increasing conceptual and methodological sophistication in how complex interventions are designed and assessed, thus offering a flexible and pluralistic guide across stages of intervention development, evaluation and implementation.^7 14 21 22^ To date, we lack a clear picture of the evolving methodological approaches used in psychosocial dementia research, and how the field has been shaped by the MRC framework. For instance, innovative approaches overcome some of the limitations of research which has traditionally used written texts in an interview format or other methods that strongly rely on abstraction, verbal communication and memory.^23^ A dementia-specific evidence synthesis is required to demonstrate how psychosocial interventions in dementia researchers design and conduct studies and whether, and how, the MRC core elements are applied in study execution. This synthesis could offer an overview of the current ‘methodological’ state of art used in psychosocial intervention studies applied research and provide valuable insights into the application of the MRC framework and its phases, areas of weakness, practical challenges and also highlight best practices. By bringing together evidence on how psychosocial dementia research is currently designed, conducted and reported, it could inform future methodological directions in psychosocial dementia-related research and may contribute to further iterations of the MRC framework.^8^

**Figure 2:**
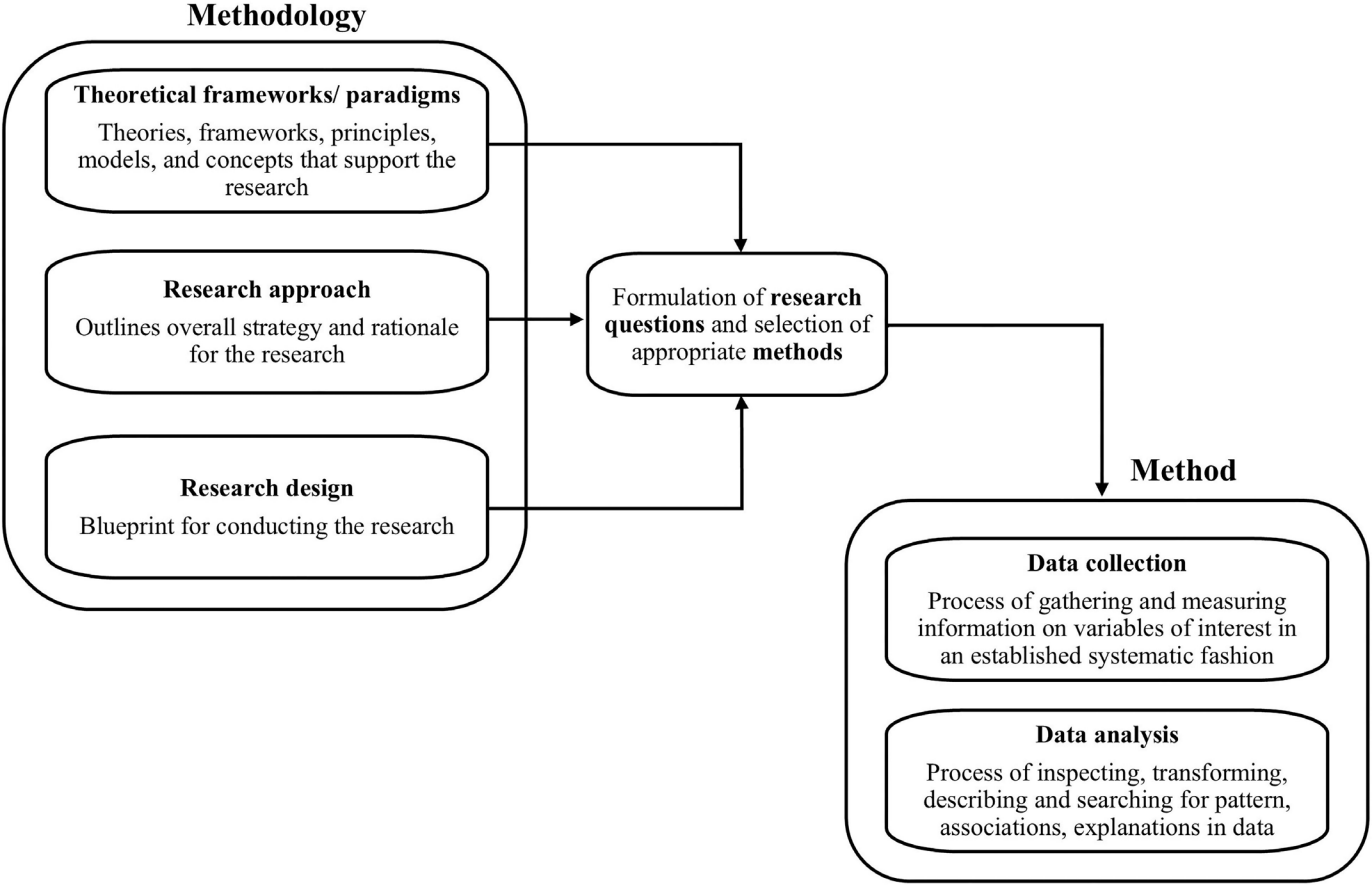
Visualisation of methodologies and methods illustrating key concepts and definitions. Note: ‘Paradigm’ was added by the authors, and data interpretation was excluded from the original graph (^24^, CC BY 4.0 licence).

The present scoping review is intended to provide a comprehensive overview of research on psychosocial interventions for dementia with a specific analytical focus on methodologies (including theoretical framework/paradigms, research approach and design) and methods (data collection and analysis)^24^ used and how the MRC core elements are covered. As the terms methodologies and methods are often used interchangeably, due to different scientific backgrounds, a definition of key terminologies is provided in figure 2.

The following research questions will be addressed:

1. Which methods and methodologies are used to develop, evaluate and/or implement psychosocial interventions for people living with dementia and unpaid carers?
2. Which methodological trends, namely an increase, maintenance or decrease in the use of specific methodologies and methods, appear between 2015–2025?
3. If and how are the core elements of the MRC framework (ie, consider context; develop, refine and (re)test programme theory; engage stakeholders; identify key uncertainties; refine intervention; economic considerations) executed in these studies per phase?
4. Which methodological gaps, such as neglected core elements, exist in psychosocial intervention dementia research?

## Methods and analysis

A scoping review approach was selected to synthesise the methodologies and methods^24^ of psychosocial interventions in dementia care. Scoping reviews aim to systematically identify and map existing evidence.^25^ Compared with other types of evidence synthesis within the ‘Big Picture’ review family—such as mapping reviews and evidence and gap maps—scoping reviews adopt an explorative, iterative and in-depth data extraction approach.^26^

The present scoping review will be guided by Arksey and O’Malley’s methodological framework^27^ which was extended and further refined.^28^ The updated framework^28^ includes: (1) defining and aligning the objectives and review questions; (2) developing and aligning the inclusion criteria with the objectives; (3) describing the planned approach to evidence searching, selection, data extraction and presentation of the evidence; (4) searching for the evidence; (5) selecting the evidence; (6) extracting the evidence; (7) analysis of the evidence; (8) presentation of the results; and (9) summarising the evidence in relation to the purpose of the review, making conclusions. This protocol adheres to the Preferred Reporting Items for Systematic reviews and Meta-Analyses extension for Scoping Reviews (PRISMA-ScR) checklist.^29^ For details, see online supplemental material 1—‘PRISMA-ScR-Checklist’. The protocol is registered under Open Science Framework (submitted https://doi.org/10.17605/OSF.IO/S56NQ).

### Identifying relevant studies

#### Eligibility criteria

The inclusion and exclusion criteria aligned with the research questions (see table 1 for details). Table 2 outlines the key definitions of the scoping review’s outcomes of interest, namely methodologies and methods, adapted from Aguira,^24^ and illustrated with dementia-specific examples. A non-exhaustive list of examples of psychosocial interventions is provided in online supplemental material 2—‘List of psychosocial interventions’.

**Table 1:** Inclusion and exclusion criteria for the present scoping review

Screening criteria	Inclusion criteria	Exclusion criteria
Language	English.	Not English.
Time	Published from 2015 to align with publication of process guidance.^7 30^	Published before 2015.
Content	Studies on psychosocial interventions for people living with dementia and unpaid carers that address the development, evaluation and/or implementation. Psychosocial interventions are defined as non-pharmacological interventions that encompass interactions between people or self-management activities, techniques, training, strategies, technology involving sensory, physical, behavioural, cognitive, emotional, interpersonal, educational, social or environmental components. These interventions can target a range of outcomes such as cognition, everyday functioning, well-being and interpersonal relationships, and range from dementia diagnosis through to palliative care, across diverse care settings. A non-exhaustive list of examples of psychosocial interventions is provided in online supplemental material 2—‘List of psychosocial interventions’.	1. Pharmacological interventions and interventions with a primary focus on: Dementia prevention. Biomedical symptom reduction. Pharmacological elements. Physiological or chemical treatment targets. AND/OR 2. Study focuses on psychometric tools, developing or evaluating scale, reviewing and discussing research methods (eg, reflective or methodological literature^58^). AND/OR 3. Intervention purely targeting physical outcomes. AND/OR 4. Interventions aimed at care workers or other stakeholders, such as training and education. AND/OR 5. Case management, care coordination or dementia advisor services, as they provide practical support, coordination and guidance to people living with dementia and unpaid carers, differ from our definition of psychosocial intervention.
Population	1. Adults aged 18 years or older living with dementia, at all stages (mild, moderate, advanced) and with these types of dementia: Alzheimer’s disease. Dementia with Lewy body. FTD including, eg, primary progressive aphasia, behavioural variant FTD^59^ and semantic dementia. Mixed dementia. Vascular dementia. AND/OR 2. Unpaid carers, referred to family members, relatives, partners/significant others, friends or neighbours. AND/OR 3. Professional care workers (health and social carer), and/or other relevant professional stakeholders. The decision to include a broad range of stakeholders reflects the understanding that key stakeholders vary depending on the context and stage of the research.	1. Animal studies. AND/OR 2. Individuals <18 years with dementia. AND/OR 3. Rarer diseases and conditions that can lead to dementia and/or dementia-like symptoms (eg, Down syndrome and dementia, posterior cortical atrophy, Parkinson’s disease dementia, encephalopathy, progressive supranuclear palsy, corticobasal syndrome, Creutzfeldt-Jakob disease, Huntington’s disease, Wernicke-Korsakoff syndrome, HIV-related dementia, alcohol-related brain injury, Korsakoff syndrome) as they often require intervention-specific approaches and distinct methodologies.
Study outcome	1. Study aims/objectives (ie, the development, feasibility/piloting, evaluation and/or implementation of a psychosocial intervention for people living with dementia and unpaid carers). AND 2. Used methodologies and methods. Methodology encompasses three key components including theoretical framework/paradigm, research approach and research design, while methods pertain to the specific technique and procedure to data collection and analysis^24^. Details of the key term definitions and dementia-specific examples are provided in table 2. AND 3. Addressed core elements as outlined in the MRC framework.^14^	Does NOT explicitly link to the development, evaluation and/or implementation of a specific psychosocial intervention.
Study design	Studies of any design according to the study aims/objectives (see above). This could include descriptive and analytical study designs, such as case reports, case series, observational (eg, cross-sectional, case–control or cohort studies), qualitative, quantitative and mixed-method designs.	N/A
Evidence source	1. Peer-reviewed study protocols and full-text articles published in English language. AND 2. Multiple publications (eg, same study) of a single intervention, if they focus on different phases (eg, development, feasibility/piloting, evaluation and/or implementation) or complementary research questions. AND 3. No geographical limitations.	1. Non-peer reviewed and grey literature, conference abstracts, posters of unpublished research, letters, commentaries, editorials, opinion pieces, guidelines, books, book chapters, reports, dissertations, monographs, as these usually lack sufficient details required by this review. AND 2. All types of knowledge synthesis reviews, including meta-analyses, as they provide limited detail on methods and methodologies used by the included studies.

FTD, frontotemporal dementia; MRC, Medical Research Council; N/A, not assessed.

**Table 2:** Definition of methodologies and methods and their concepts adapted from Aguira,^24^ alongside dementia-specific examples

Terminology	Specific terms	Definition	Dementia-specific examples
Methodology: approach or framework used in research, influenced by the researcher’s theoretical perspective and guiding principles; encompasses researcher’s beliefs, theories and values that support their approach^60^	Theoretical framework paradigm	Theories,* concepts,† principles,‡ frameworks§ and models¶ that support the research per phases^24^	‘We used the updated Framework for Reporting Adaptations and Modifications-Expanded to report adaptations made on the Care Of People with dementia in their Environments (COPE) program to be translated into residential aged care.’**^61^
	Research design	Blueprint for conducting the research^24^	‘The design of this study was based on an open-label pilot randomized controlled trial, as it was not possible to blind the intervention to participants, therapists, and assessor.’^62^ ‘A hybrid effectiveness implementation feasibility study was undertaken to evaluate the implementation process and explore program outcomes from the delivery of AoP@Home for people with dementia and their unpaid carers living in the community.’^63^
	Research approach	Outlines overall strategy (including coapproaches) and rationale for the research^24^	‘For this, we first drew on the ‘planning’ and ‘design’ phases of the Person-Based Approach (PBA) for developing behavioral interventions. We then incorporated the principles of Patient and Public Involvement (PPI) in health and medical research by using a co-design process that broadly aligned with the ‘deciding how to do it’/‘designing and managing’ phase of the research cycle.’**^64^
Method: systematic techniques, procedures or tools practically employed for data collection, analysis and interpretation^60^	Data collection	Process of gathering and measuring information on variables of interest in an established systematic fashion^24^	‘This study was a cross-sectional design. Observational data were used from questionnaires and interviews that were obtained (…) of the Living Arrangements for Dementia (LAD) study.’^65^
	Data analysis	Process of inspecting, transforming, describing and searching for pattern, associations, explanations in data^66^	‘A pre- and post- comparison was completed for participant specific outcomes. (…)All interviews and the focus group discussion were audio-recorded, transcribed verbatim by a third-party transcription service. The primary analysis was complete by the first study author (MR) using a thematic approach.’**^61^ ‘The current study reports a qualitative exploration of the experiences of key stakeholders involved in the implementation of AoP@Home, using qualitative content analysis of interview data that was collected during the feasibility study.’^63^

We acknowledge that the following definitions may vary within and across disciplines:

* Theory is ‘a set of inter-related concepts, definitions and propositions that present a systematic view of events or situations by specifying relations among variables, to explain and predict the events or situations. The notion of generality, or broad application, is important’.^67^

† Concepts are the ‘building blocks of theory or the primary elements’. A theory is made up of concepts.^67^

‡ Principles are grounded in theories and concepts.^68^

§ Frameworks are structures for presenting concepts, without necessarily preserving inter-relationships between individual concepts.^67^

¶ Models are ‘a generalised or hypothetical description used to analyse or explain something’. Increasingly, *logic models* are used to explain how an intervention is thought to work.^67 69^

** In-text references were not included here, but can be found in the original articles.

#### Search strategy

The systematic search will identify all relevant studies from the year 2015 onwards, when process (ie, *how to*) guidance^30^ for the MRC framework, relevant to our research, first appeared.^7^ The following electronic databases will be searched for eligible studies:

- Ovid MEDLINE.
- Embase.
- PsycINFO.
- CINAHL.
- Web of Science.
- Cochrane Library.

The search strategy tailored to each database was developed and refined in collaboration with the library support services from Maastricht University and Karolinska Institutet in January and February 2025. Search terms include keywords and Medical Subject Headings terms representing three main concepts consistent with the Population, Concept, Context framework.^28^ These include dementia (Population), psychosocial intervention (Concept) and develop, feasibility/pilot, evaluate and implement phase (Context) (online supplemental material 3—‘Documentation of search strategies’). The search will be performed by the library support service team from Karolinska Institutet, including de-duplication. Only studies published in English will be included^31 32^ due to the limited translation resources and the expected large volume of retrieved articles. The screening process is expected to commence in March 2025 and the study to be complete by July 2026.

### Artificial intelligence data management

This scoping review will use two artificial intelligence (AI) tools to support the screening and selection process: Covidence and ASReview. The integration of these tools provides several advantages such as (1) enabling formulation and addressing research questions for synthesis of a large number of articles; (2) significantly reducing the screening and selection workload by 20–54%^33^,^35^; and (3) minimising the risk of bias in the screening and selection process.^36^

Covidence is a machine learning software, which specifically is designed for reference management and supports independent screening.^37^ It was selected from other available tools (eg, Abstrackr, Colandr, Rayyan, DRAGON, EPPI-Reviewer) as it facilitates independent, blinded screening by multiple reviewers, ranks studies that are most likely to be included based on the reviewers’ past screening decision and enables the creation of custom labels for study exclusion.^35 38^ ASReview^39 40^ will complement the screening process by performing semi-automated screening of title and abstract of the records. This tool employs an active researcher-in-the-loop machine learning algorithm to rank articles based on their probability of relevance.^36^ The algorithm uses a pool of labelled articles (‘relevant’ and ‘irrelevant’) to refine its ranking over time.^39^ To ensure reliability, accuracy and reduce the risk of AI-induced biases such as overlooking relevant studies, a check validation activity will be used.^41 42^ Below, we provide detailed descriptions of the screening and selection process, as well as the specific roles of these AI tools and the check validation activity.

### Screening and selecting studies for inclusion

**Figure 3:**
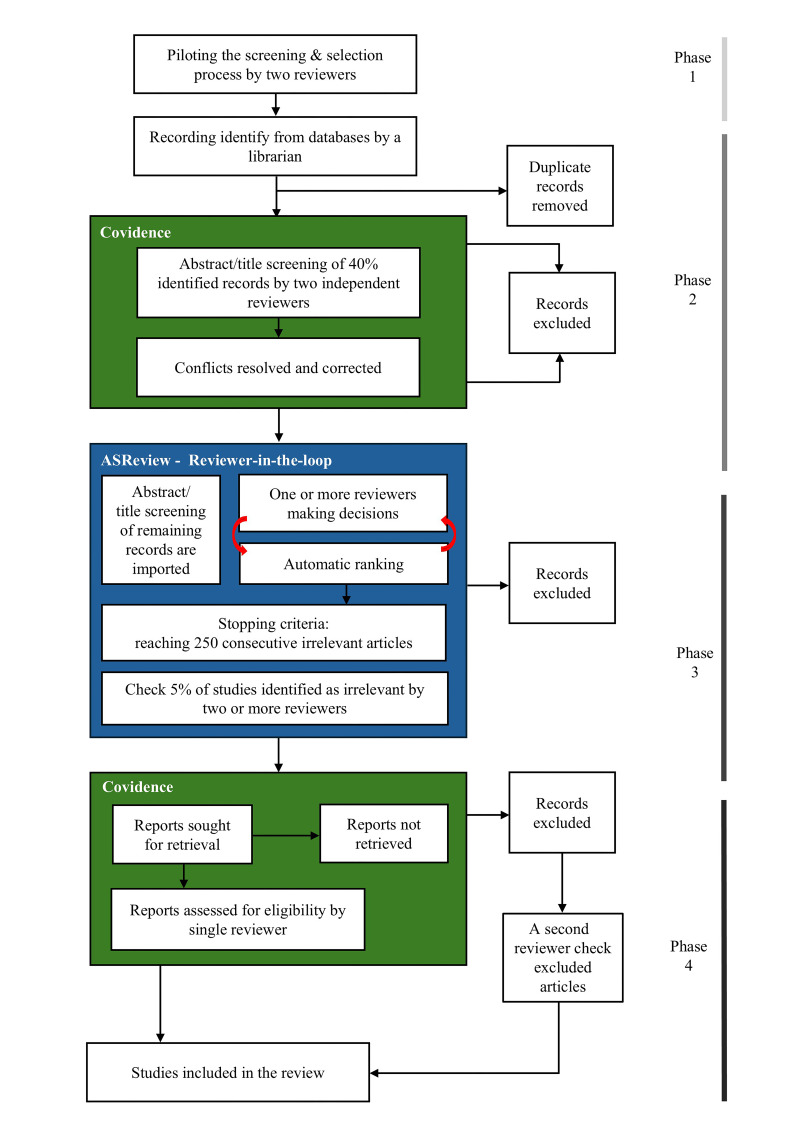
Summary of the screening and selection process.

The screening and study selection process includes four stages (figure 3).

Stage 1: Two independent reviewers (FD’A, SLB) will conduct a pilot test of the screening process in January 2025, using a subset of 20 articles retrieved from the MEDLINE database. These will be uploaded to Covidence^37^ and independently screened as ‘include’, ‘exclude’ or ‘maybe’ based on predefined inclusion criteria. Any discrepancies will be resolved through discussion between two reviewers (FD’A, SLB), and in case of disagreement, a third author (MG). This stage helps to refine the inclusion and exclusion criteria, ensures consistency of the approach taken in the study selection process, and facilitates the creation of a list of exclusion reason labels to be used across reviewers in the screening process.^43^

Stage 2: The librarian from Karolinska Institutet will conduct the search strategy on the selected databases and remove duplication. The deduplicated results of the search strategy will be imported into Covidence.^37^ Two independent reviewers (coauthors) will screen titles and abstracts of up to 40% of the total records identified.^36^ If the eligibility of a study is unclear from the abstract, it will be marked as ‘maybe’. Studies assessed as not eligible will be excluded, with reasons documented in the software. Any discrepancies will be resolved through consensus meetings between the reviewers (FD’A, SLB, ANB), and potentially another member of the review team (MG) if no agreement can be reached. Results of the screening process from this stage will be used to train the ASReview^39^ algorithm in Stage 3.^36^

Stage 3: Once consensus is reached, search results will be imported into ASReview.^39^ The screening decision (‘relevant’ or ‘irrelevant’) for the initial up to 40% of articles will be used to train the algorithm and then the first ranking of all unlabeled articles will be conducted. Reviewers (FD’A, SLB, ANB) will sequentially screen title and abstract of the highest-ranked articles, making decisions against the eligible criteria. This process (AI ranking and reviewer making decision) will be repeated until a predefined data-driven stopping criteria is reached. To ensure a reliable approach, our review will set the data-driven stopping criteria at up to 250 consecutive irrelevant articles, considerably above the average stopping criteria (ie, 50 or 100 articles).^36^ Up to 5% of studies identified as irrelevant by the ASReview algorithm will be checked against inclusion criteria by an independent reviewer (FD’A, SLB).

Stage 4: Studies screened as ‘eligible’ will be uploaded into Covidence. The full texts of these included records during stage two and three will be retrieved and screened by one reviewer (coauthors). A second independent reviewer (FD’A) will check those articles that have been excluded in the full-text screening. All sources of evidence evaluated at the full-text stage that do not meet the inclusion criteria will be documented, with the reasons for exclusion reported in the PRISMA flow diagram.^44^,^46^

### Data extraction

Data extraction of all included studies will be performed by two or more reviewers (coauthors) using a data extraction form. This form will be piloted by two review team members on a sample of ten studies to ensure its accuracy and ability to capture all relevant data comprehensively.^43^ The data extraction form will include (1) general study information (eg, author, year, funding, country, publication type, sample characteristics, setting (eg, care homes, hospitals), sector (public, private or third sector encompassing community, voluntary and social enterprise), outcome measures, intervention type, study aim/objective, research question), (2) study phase (ie, development, feasibility, piloting, evaluation, implementation), (3) methodology (eg, framework/ paradigm, design, approach) and (4) method (eg, data collection, data analysis) and (5) information on whether and which of the MRC six core elements have been addressed (yes/no) and qualitative details on how they were addressed. The data extraction form will be further refined during the data extraction process, if needed.^43^ As this scoping review aims at synthesising methodological approaches, no risk of bias or quality assessments of the included studies will be performed. This is consistent with synthesis review studies with similar aims and guidelines for conducting scoping reviews.^28 43^

### Data synthesis

Given the methodological focus of the review and broad range of our research questions, narrative synthesis^15^ alongside visual approaches^16^ will be used to capture the knowledge, identify trends and highlight critical methodological gaps. Studies will be categorised in terms of the objectives of the scoping review, such as methodologies and methods used within the MRC framework phases (ie, develop/adapt/identify intervention; feasibility/piloting, evaluation, implementation) and whether its six core elements were addressed. This synthesis will provide a comprehensive overview of the methodologies and methods in psychosocial interventions in dementia care in the past ten years and enable identification of methodological trends and gaps across studies. Trends will be determined by using percentages and graphical representations (eg, table, bar charts and histograms) to quantify and depict knowledge clusters such as numbers of studies that used certain methodology or method over time, across geographical regions, within specific types of psychosocial interventions and participant social cultural demographic, or stage of dementia. To support the identification of trends, summary and presentation of the key findings, software such as EviAtlas will be used for producing clear and interactive graphical visualisations.^47^

## Ethics and dissemination

The scoping review methodology consists of reviewing and collecting data from publicly available literature; therefore, this protocol does not require ethics approval. To our knowledge, this scoping review will be the first to provide a comprehensive overview of methodologies and methods used in the development, evaluation and/or implementation of psychosocial interventions for people living with dementia and unpaid carers. The findings have the potential to advance dementia research by synthesising the current practice, identifying trends, methodologies and methods used, and highlighting gaps with a particular focus on one or more of the four key phases outlined in the MRC framework (ie, development, feasibility/piloting, evaluation and implementation), and related core elements.

The study dissemination will be multimodal, targeting a range of audiences ranging from dementia researchers, methodologists, funding bodies, policymakers, dementia charities, clinicians, people living with dementia and carers. The results will be disseminated through peer-reviewed publication(s), seminars, webinars and conferences where we anticipate opportunities for discussion and debate. Wider dissemination of results will be conducted through education at relevant master programmes at universities in different countries, writing blogs, lay summaries published in social media, commissioner funder briefing and presentations at local community events involving people living with dementia and carers.

Notwithstanding the contributions this review can make, there are some limitations. Only studies published in English will be included.^31 32^ A range of evidence sources will be excluded, such as letters, commentaries, editorials, opinion pieces, guidelines, books, book chapters, reports, dissertations and monographs, since our focus is essentially on peer-reviewed outlines of psychosocial intervention studies. Despite these potential constraints, by highlighting existing gaps, this review will feed into a Delphi consensus study, to elicit opinions to reach consensus on (1) relevance of core elements when developing, evaluating and implementing psychosocial interventions in dementia and (2) methodologies and methods most suitable per phase. The Delphi study will involve the scientific community, people with lived experiences of dementia, professional health and social care workers and other stakeholders (eg, funding bodies, policy makers) (ethical approval reference: Maastricht University FHML-REC/2025/078 and the University of West London UWL/REC/SBS-01195). Feasibility of addressing core elements and using certain methodologies and methods will also be explored. Both the scoping review and the Delphi study will provide valuable insights into the methodologies and methods in psychosocial interventions dementia research that may inform future iterations of the MRC framework, thus leading to more efficient, practical and impactful research and practice.

### Review status

The reviewers have commenced searching relevant studies on the electronic databases on 28 March 2025. This review is expected to be complete by July 2026.

### Patient and public involvement

It was not appropriate to involve patients and the public in this scoping review. However, the outcomes of this scoping review will inform a Delphi study as part of the METHODEM project (‘METHOdological consensus for complex interventions in DEMentia’),^8^ in which an advisory group consisting of people living with dementia, carers and healthcare professionals as well as a multidisciplinary steering committee (ie, the INTERDEM Methodology Taskforce (early, timely and quality psychosocial INTERventions in DEMentia)) will be involved to ensure societal relevance and feasibility.

## Supplementary material

10.1136/bmjopen-2025-114584 online supplemental file 1

10.1136/bmjopen-2025-114584 online supplemental file 2

10.1136/bmjopen-2025-114584 online supplemental file 3
