# Supplementary material for: Methodologies and methods for the development, evaluation and implementation of psychosocial interventions for dementia: protocol for a scoping review
Source: BMJ Open. 2026 Apr 24;16(4):e114584. doi: 10.1136/bmjopen-2025-114584 (PMC13110686; doi:10.1136/bmjopen-2025-114584)
Supplement: online supplemental file 2 [file bmjopen-16-4-s002.docx]

**Methodologies and methods for the development, evaluation, and implementation of psychosocial interventions for dementia: Protocol for a scoping review**

**Examples of psychosocial interventions in dementia care within sensory, cognitive, physical, behavioural, educational, emotional, social, and/or environmental domains.**

Please note that this list of examples is not intended to be comprehensive.

| **Domains** | **Example of interventions** |
| --- | --- |
| Sensory, cognitive, physical, behavioral, educational, emotional, social, and/or environmental | Acoustic stimulation |
|  | Anger management therapy / Anger management |
|  | Applied behavior analysis / Functional analysis / behaviour management |
|  | Aromatherapy |
|  | Art therapy / intervention / programme / activity |
|  | Artificial intelligence (AI)-supported, chat-bot |
|  | Aversive therapy |
|  | Bereavement / Grief therapy |
|  | Bibliotherapy |
|  | Care planning |
|  | Cognitive rehabilitation |
|  | Cognitive remediation |
|  | Cognitive restructuring |
|  | Cognitive stimulation therapy / Maintenance cognitive stimulation therapy |
|  | Cognitive training |
|  | Color therapy |
|  | Commitment therapy |
|  | Communication training |
|  | Compassion-focused therapy |
|  | Compensatory strategies e.g. aides / colour |
|  | Counselling |
|  | Creative writing (poetry, prose, fiction to screen and stage writing, including memoirs and personal reflective pieces) |
|  | Dancing / dance therapy / intervention / programme |
|  | Dementia / memory / Alzheimer cafés |
|  | Dementia choirs / singing / ‘singing for the brain groups’ |
|  | Dementia-friendly community initiative / programme / hub |
|  | Dialectical behavior therapy |
|  | Dignity therapy |
|  | Dog therapy |
|  | Doll / doll therapy |
|  | Drama therapy / Psychodrama / Role playing |
|  | End of life intervention |
|  | Environment design and construction |
|  | Equine-Assisted therapy |
|  | Facility design e.g. gardens / space |
|  | Family therapy |
|  | Forest therapy |
|  | Grief therapy |
|  | eHealth/ mHealth |
|  | Horticultural therapy / intervention / programme |
|  | Hydration care |
|  | Involvement groups for people with dementia |
|  | Life review / Life review therapy |
|  | Life story work |
|  | Meeting centres programme |
|  | Mental Healing |
|  | Mindfulness |
|  | Movement therapy / intervention / programme |
|  | Multi-sensory intervention / programme |
|  | Museum based-programme / intervention |
|  | Music / music therapy / intervention / programme |
|  | Namaste |
|  | Nutritional care |
|  | Object handling intervention / programme |
|  | Pain management |
|  | Palliative interventions |
|  | Participatory arts |
|  | Pet therapy |
|  | Person-centered psychotherapy |
|  | Photo voice intervention |
|  | Play therapy |
|  | Psychoanalytic therapy |
|  | Psychoeducation |
|  | Reality orientation |
|  | Reflexology |
|  | Relaxation therapy |
|  | Reminiscence therapy / intervention / programme |
|  | (Social/ anomalopid/ pet) robots/ robotics |
|  | Smart homes/ devices |
|  | Snoezelen |
|  | Social media |
|  | Social activities |
|  | Strength and balance |
|  | Systemic therapy |
|  | Tai Ji |
|  | Talking therapy |
|  | Tele-health/ -rehabilitation/ -care |
|  | Theatre-mediated intervention / programme |
|  | Therapeutic touch |
|  | Validation therapy |
|  | Video-/tele-conferencing and consulting/ messaging/ SMS/ email |
|  | Video/ computer games |
|  | Virtual reality/ virtual world experience |
|  | Walking group programme |
|  | Wearables/ sensors/ smartwatches |
|  | Web-/ internet-based/ online/ digital/ website |
|  | Wii/ Exergaming program |

Note: The psychosocial interventions listed above can be delivered by a range of professionals in accordance with certification requirements, including arts therapists, massage therapists, occupational therapists, physiotherapists, psychologists, social workers, speech and language therapists, recreational therapists, or reflexologists.
