# Supplementary material for: Methodologies and methods for the development, evaluation and implementation of psychosocial interventions for dementia: protocol for a scoping review
Source: BMJ Open. 2026 Apr 24;16(4):e114584. doi: 10.1136/bmjopen-2025-114584 (PMC13110686; doi:10.1136/bmjopen-2025-114584)
Supplement: online supplemental file 3 [file bmjopen-16-4-s003.pdf]

Methodologies and methods for the development, evaluation, and implementation of psychosocial interventions for dementia: Protocol for a scoping review

Documentation of search strategies

A literature search was performed in the following databases: Medline (Ovid), Embase (embase.com), Cochrane Library (Wiley), Web of Science (Clarivate Analytics), PsycINFO (EBSCOhost) and CINAHL (EBSCOhost). The last search was conducted 2025-03-28.

The search strategy was developed in Medline (Ovid) in collaboration with librarians at the Karolinska Institutet University Library. For each search concept Medical Subject Headings (MeSH-terms) and free text terms were identified. The search was then translated, in part using Polyglot Search Translator (1), into the other databases.

Language restriction was made to English.

Articles older than 2015 were not reviewed.

The strategies were peer reviewed by another librarian prior to execution.

1. Medline

Interface: **Ovid MEDLINE(R) ALL** content coverage from 1946<br><br>Date of Search: 28 March 2025<br><br>Number of hits: 8,044<br><br>Comment: In Ovid, two or more words are automatically searched as phrases; i.e. no quotation marks are needed	Field labels<br><br>• exp/ = exploded MeSH term<br>• / = non exploded MeSH term<br>• .ti,ab,kf. = title, abstract and author keywords<br>• adjx = within x words, regardless of order<br>• * = truncation of word for alternate endings<br>• ? = 0-1 letter/number<br>• # = 1 letter/number

Database(s): **Ovid MEDLINE(R) ALL** 1946 to March 27, 2025
Search Strategy:

#	Searches	Results
1	Dementia/	67122
2	Alzheimer Disease/	132194
3	Lewy Body Disease/	4777
4	Frontotemporal Dementia/	5129
5	Mixed Dementias/	12
6	Dementia, Vascular/	5967
7	Aphasia, Primary Progressive/	1180
8	(alzheimer* or amentia* or benson* syndrome* or binswanger* disease* or binswanger* encephalopath* or dementia* or familial pick* disease* or lewy body disease* or mesulam* syndrome* or posterior cortical atroph* or primary progressive aphasia* or subcortical leukoencephalopath* or wilhelmsen lynch disease*).ti,ab,kf.	316202
9	or/1-8	336439
10	exp Psychotherapy/	230333
11	Laughter Therapy/	287
12	Mental Healing/	1516
13	Forest Therapy/	0
14	Therapeutic Touch/	1025
15	Massage/	7115
16	Reflexotherapy/	470
17	exp Sensory Art Therapies/	57856
18	exp Spiritual Therapies/	16385
19	Occupational Therapy/	15821
20	Therapy Animals/	40
21	Singing/	1503
22	Self-Help Groups/	9799
23	exp Social Support/	84835
24	Advance Care Planning/	4776
25	Palliative Care/	66955
26	exp Environment Design/	9120
27	exp "Facility Design and Construction"/	26552

28	exp Environment, Controlled/	364054
29	(aromatherap* or bibliotherap* or chromatotherap* or chromotherap* or dramatherap* or ecotherap* or psychotherap* or reflexotherap*).ti,ab,kf.	61365
30	((caregiver or complementary or family or group or narrative or occupational or play) adj (intervention* or program* or therap* or treatment*)).ti,ab,kf.	55395
31	(crisis intervention* or self-help group* or sensitivity training group*).ti,ab,kf.	4259
32	(("acceptance and commitment" or acoustic stimulation or anger management or applied behavio?r analys#s or aroma or aversion or auditory stimulation or aversive or behavior-change or client centered or clown or colo?r or compassion* or communication training or compensatory strateg* or conditioning or conversation* coaching or coping or culture-based or couple or dialectical behavio?r* or dignity or directed reverie or doll? or drama or emotion* focused or encounter group* or forest or free association* or garden* or gestalt or grief or guided imagery or health educat* or healing or horticultur* or laughter or life review or laying-on-of-hands or life story work or marital or marriage or massage or meditation or mindfulness or mixed realit* or multi family or multifamily or multiple family or multisensory or multi-sensory or namaste care or nature or nondirective or object handling or peer group* or personal validation or photo-voice or play-based mental health or psychoanalytic* or psychodrama or psycholog* or psychosocial* or psycho-social* or reablement or reality or reflex or reiki or relaxation or reminiscence or rogerian or role playing* or sandplay or sensory stimulation or shinrin-yoku or snoezelen or social support or socioenvironment* or socio-environment* or solution focused brief or t-group* or talking or touch*) adj2 (intervention* or program* or therap* or treatment*)).ti,ab,kf.	88019
33	(acoustic stimulation or anger management or applied behavio?r analys#s or aroma or auditory stimulation or choir* or communication training or conversation* coaching or compensatory strateg* or clown? or creative writing or doll? or drama or emotion* focused or encounter group* or forest bath* or free association* or garden* or grief counseling or guided imagery or horticultur* or laughter or handicraft* or laying-on-of-hands or life story work or massage or meditation or mindfulness or mixed realit* or multisensory stimulation or museum* or music or namaste care or peer group* or personal validation or photo-voice or poetry or psychodrama or reablement or reality orientation or reiki or relaxation techni* or reminiscence or role playing* or sandplay or sensory stimulation or shinrin-yoku or snoezelen or singing or storytelling or systematic therap* or t-group* or theatre?).ti,bt,kf.	71363
34	((handicraft* or poetry or reading or singing or storytelling or theatre?) adj2 (activit* or intervention* or program* or therap* or treatment*)).ti,ab,kf.	2597
35	((cognition or cognitive or cognitive behavio*) adj1 (intervention* or program* or rehabilitation or reframing or remediation or restructuring or therap* or training)).ti,ab,kf.	16981
36	((health educat* or nonpharmacolog* or non-pharmacolog*) adj2 (intervention* or program* or therap* or treatment*)).ti,ab,kf.	23973
37	((art or choir* or music or writing) adj2 (activit* or alzheimer* or dementia* or making or intervention* or participat* or program* or therap* or treatment*)).ti,ab,kf.	34925
38	(behavio* adj (change technique* or counsel* or modification* or therap* or treatment* or support)).ti,ab,kf.	50650
39	(museum* adj2 (activit* or attend* or alzheimer* or dementia* or intervention* or participat* or program* or therap* or visit)).ti,ab,kf.	128
40	(community adj2 (activit* or hub? or initiative* or intervention* or program*)).ti,ab,kf.	27039
41	((alzheimer* or dementia* or memory) adj2 cafe?).ti,ab,kf.	37
42	(social interaction intervention* or social intervention*).ti,ab,kf.	1501

43	(social activit* adj2 (intervention* or program* or therap* or treatment*)).ti,ab,kf.	105
44	(meeting centre* adj2 program*).ti,ab,kf.	17
45	((animal* or animal assisted or dog? or equine* or pet? or pet assisted) adj2 (emotional support or intervention* or therap*)).ti,ab,kf.	6802
46	((assistive or companion*) adj2 (animal? or pet?)).ti,ab,kf.	5493
47	((animal* or human* or pet? or social) adj2 robot*).ti,ab,kf.	4816
48	((care planning or end of life or psychoeducation* or psycho-education* or palliative care) adj2 (intervention* or program* or therap* or treatment*)).ti,ab,kf.	9041
49	((architectural or environmental* or facility or facilities or garden* or human centered or residential or universal) adj2 design*).ti,ab,kf.	6484
50	(built environment* or environment* adaptation*).ti,ab,kf.	10180
51	(home* adj2 modification*).ti,ab,kf.	621
52	or/10-51	1130618
53	User-Centered Design/	326
54	Program Evaluation/	68916
55	Evaluation Studies as Topic/	122485
56	Evaluation Study/	265426
57	Feasibility Studies/	90545
58	Implementation Science/	1683
59	Pilot Projects/	158924
60	Comparative Effectiveness Research/	4121
61	exp "Costs and Cost Analysis"/	277468
62	exp Clinical Trial/	1019297
63	(acceptability or adapt* or adherence or adoption* or advantage* or appropriateness or barrier? or business model* or cost? or create or customis* or customiz* or deliver or design* or develop* or document* or effect? or effectiveness or efficacy or evaluat* or exploratory or facilitator* or feasibility or fidelity or impact* or implement* or "mechanism* of change" or penetration or pilot or plan or produce or production* or proof-of-concept or quality or refine or sustainability or testing or theory or theoretical or theories or trial? or trialability or uncertainty or uptake* or usability or user-centered).ti,ab,kf.	20221461
64	or/53-63	20537749
65	9 and 52 and 64	13168
66	65 not (animals not humans).sh.	12944
67	limit 66 to yr="2015 -Current"	8316
68	limit 67 to english language	8044

2. Embase

Interface: **embase.com** content coverage from 1947<br><br>Date of Search: 28 March 2025<br><br>Number of hits: 10,172<br><br>Comment: Emtree is the controlled vocabulary in Embase	Field labels<br><br>• /exp = exploded Emtree term<br>• /de = non exploded Emtree term<br>• ti,ab,kw = title, abstract and author keywords<br>• NEAR/x = within x words, regardless of order<br>• * = truncation of word for alternate endings<br>• $ = 0-1 letter/number<br>• ? = 1 letter/number

No.	Query	Results
#69	#66 NOT #67 AND [english]/lim	10172
#68	#66 NOT #67	10520
#67	#66 AND ('Conference Abstract'/it OR 'Conference Review'/it OR 'Editorial'/it OR 'Letter'/it)	3341
#66	#64 NOT ([animals]/lim NOT [humans]/lim) AND [2015-2025]/py	13861
#65	#64 NOT ([animals]/lim NOT [humans]/lim)	21484
#64	#9 AND #54 AND #63	22030
#63	#55 OR #56 OR #57 OR #58 OR #59 OR #60 OR #61 OR #62	25960636
#62	acceptability:ti,ab,kw OR adapt*:ti,ab,kw OR adherence:ti,ab,kw OR adoption*:ti,ab,kw OR advantage*:ti,ab,kw OR appropriateness:ti,ab,kw OR barrier$:ti,ab,kw OR 'business model*':ti,ab,kw OR cost$:ti,ab,kw OR create:ti,ab,kw OR customis*:ti,ab,kw OR customiz*:ti,ab,kw OR deliver:ti,ab,kw OR design*:ti,ab,kw OR develop*:ti,ab,kw OR document*:ti,ab,kw OR effect$:ti,ab,kw OR effectiveness:ti,ab,kw OR efficacy:ti,ab,kw OR evaluat*:ti,ab,kw OR exploratory:ti,ab,kw OR facilitator*:ti,ab,kw OR feasibility:ti,ab,kw OR fidelity:ti,ab,kw OR impact*:ti,ab,kw OR implement*:ti,ab,kw OR 'mechanism* of change':ti,ab,kw OR penetration:ti,ab,kw OR pilot:ti,ab,kw OR plan:ti,ab,kw OR produce:ti,ab,kw OR production*:ti,ab,kw OR 'proof of concept':ti,ab,kw OR quality:ti,ab,kw OR refine:ti,ab,kw OR sustainability:ti,ab,kw OR testing:ti,ab,kw OR theory:ti,ab,kw OR theoretical:ti,ab,kw OR theories:ti,ab,kw OR trial$:ti,ab,kw OR trialability:ti,ab,kw OR uncertainty:ti,ab,kw OR uptake*:ti,ab,kw OR usability:ti,ab,kw OR 'user centered':ti,ab,kw	25580051
#61	'clinical trial'/exp	2009961
#60	'comparative effectiveness'/de	155014
#59	'pilot study'/de	235335
#58	'implementation science'/de	7605
#57	'feasibility study'/de	205277
#56	'evaluation study'/de	238778
#55	'program evaluation'/exp	40271
#54	#10 OR #11 OR #12 OR #13 OR #14 OR #15 OR #16 OR #17 OR #18 OR #19 OR #20 OR #21 OR #22 OR #23 OR #24 OR #25 OR #26 OR #27 OR #28 OR #29 OR #30 OR #31 OR #32 OR #33 OR #34 OR #35 OR #36 OR #37 OR #38 OR #39 OR #40 OR #41 OR #42 OR #43 OR #44 OR #45 OR #46 OR #47 OR #48 OR #49 OR #50 OR #51 OR #52 OR #53	1115437
#53	(home* NEAR/2 modification*):ti,ab,kw	863
#52	'built environment*':ti,ab,kw OR 'environment* adaptation*':ti,ab,kw	10673

#51	((architectural OR environmental* OR facility OR facilities OR garden* OR 'human centered' OR residential OR universal) NEAR/2 design*):ti,ab,kw	8469
#50	(('care planning' OR 'end of life' OR 'psycho education*' OR psychoeducation* OR 'palliative care') NEAR/2 (intervention* OR program* OR therap* OR treatment*)):ti,ab,kw	14116
#49	((animal* OR human* OR pet$ OR social) NEAR/2 robot*):ti,ab,kw	4278
#48	((assistive OR companion*) NEAR/2 (animal$ OR pet$)):ti,ab,kw	6208
#47	((animal* OR 'animal assisted' OR dog$ OR equine* OR pet$ OR 'pet assisted') NEAR/2 ('emotional support' OR intervention* OR therap*)):ti,ab,kw	10099
#46	('meeting centre*' NEAR/2 program*):ti,ab,kw	28
#45	('social activit*' NEAR/2 (intervention* OR program* OR therap* OR treatment*)):ti,ab,kw	136
#44	'social interaction intervention*':ti,ab,kw OR 'social intervention*':ti,ab,kw	1973
#43	((alzheimer* OR dementia* OR memory) NEAR/2 cafe$):ti,ab,kw	44
#42	(community NEAR/2 (activit* OR hub$ OR initiative* OR intervention* OR program*)):ti,ab,kw	34392
#41	(museum* NEAR/2 (activit* OR attend* OR alzheimer* OR dementia* OR intervention* OR participat* OR program* OR therap* OR visit)):ti,ab,kw	145
#40	(behavio* NEXT/1 ('change technique*' OR counsel* OR modification* OR therap* OR treatment* OR support)):ti,ab,kw	69847
#39	((art OR choir* OR music OR writing) NEAR/2 (activit* OR alzheimer* OR dementia* OR making OR intervention* OR participat* OR program* OR therap* OR treatment*)):ti,ab,kw	49553
#38	(('health educat*' OR nonpharmacolog* OR 'non pharmacolog*') NEAR/2 (intervention* OR program* OR therap* OR treatment*)):ti,ab,kw	32216
#37	((cognition OR cognitive OR 'cognitive behavio*') NEAR/1 (intervention* OR program* OR rehabilitation OR reframing OR remediation OR restructuring OR therap* OR training)):ti,ab,kw	59917
#36	((handicraft* OR poetry OR reading OR singing OR storytelling OR theatre$) NEAR/2 (activit* OR intervention* OR program* OR therap* OR treatment*)):ti,ab,kw	3276
#35	'acoustic stimulation':ti,kw OR 'anger management':ti,kw OR 'applied behavio$r analys?s':ti,kw OR aroma:ti,kw OR 'auditory stimulation':ti,kw OR choir*:ti,kw OR 'communication training':ti,kw OR 'conversation* coaching':ti,kw OR 'compensatory strateg*':ti,kw OR clown$:ti,kw OR 'creative writing':ti,kw OR doll$:ti,kw OR drama:ti,kw OR 'emotion* focused':ti,kw OR 'encounter group*':ti,kw OR 'forest bath*':ti,kw OR 'free association*':ti,kw OR garden*:ti,kw OR 'grief counseling':ti,kw OR 'guided imagery':ti,kw OR horticultur*:ti,kw OR laughter:ti,kw OR handicraft*:ti,kw OR 'laying on of hands':ti,kw OR 'life story work':ti,kw OR massage:ti,kw OR meditation:ti,kw OR mindfulness:ti,kw OR 'mixed realit*':ti,kw OR 'multisensory stimulation':ti,kw OR museum*:ti,kw OR music:ti,kw OR 'namaste care':ti,kw OR 'peer group*':ti,kw OR 'personal validation':ti,kw OR 'photo voice':ti,kw OR poetry:ti,kw OR psychodrama:ti,kw OR reablement:ti,kw OR 'reality orientation':ti,kw OR reiki:ti,kw OR 'relaxation techni*':ti,kw OR reminiscence:ti,kw OR 'role playing*':ti,kw OR sandplay:ti,kw OR 'sensory stimulation':ti,kw OR 'shinrin yoku':ti,kw OR snoezelen:ti,kw OR singing:ti,kw OR storytelling:ti,kw OR 'systematic therap*':ti,kw OR 't group*':ti,kw OR theatre$:ti,kw	81807
#34	(('acceptance and commitment' OR 'acoustic stimulation' OR 'anger management' OR 'applied behavio$r analys?s' OR aroma OR aversion OR 'auditory stimulation' OR aversive OR 'behavior change' OR 'client centered' OR clown OR colo$r OR compassion* OR 'communication training' OR 'compensatory strateg*' OR	118048

	conditioning OR 'conversation* coaching' OR coping OR 'culture based' OR couple OR 'dialectical behavio$r*' OR dignity OR 'directed reverie' OR doll$ OR drama OR 'emotion* focused' OR 'encounter group*' OR forest OR 'free association*' OR garden* OR gestalt OR grief OR 'guided imagery' OR 'health educat*' OR healing OR horticultur* OR laughter OR 'life review' OR 'laying on of hands' OR 'life story work' OR marital OR marriage OR massage OR meditation OR mindfulness OR 'mixed realit*' OR 'multi family' OR multifamily OR 'multiple family' OR multisensory OR 'multi sensory' OR 'namaste care' OR nature OR nondirective OR 'object handling' OR 'peer group*' OR 'personal validation' OR 'photo voice' OR 'play-based mental health' OR psychoanalytic* OR psychodrama OR psycholog* OR psychosocial* OR 'psycho social*' OR reablement OR reality OR reflex OR reiki OR relaxation OR reminiscence OR rogerian OR 'role playing*' OR sandplay OR 'sensory stimulation' OR 'shinrin yoku' OR snoezelen OR 'social support' OR socioenvironment* OR 'socio environment*' OR 'solution focused brief' OR 't group*' OR talking OR touch*) NEAR/2 (intervention* OR program* OR therap* OR treatment*)):ti,ab,kw	
#33	'crisis intervention*':ti,ab,kw OR 'self-help group*':ti,ab,kw OR 'sensitivity training group*':ti,ab,kw	5703
#32	((caregiver OR complementary OR family OR group OR narrative OR occupational OR play) NEXT/1 (intervention* OR program* OR therap* OR treatment*)):ti,ab,kw	81530
#31	aromatherap*:ti,ab,kw OR bibliotherap*:ti,ab,kw OR chromatotherap*:ti,ab,kw OR chromotherap*:ti,ab,kw OR dramatherap*:ti,ab,kw OR ecotherap*:ti,ab,kw OR psychotherap*:ti,ab,kw OR reflexotherap*:ti,ab,kw	87038
#30	'sensory stimulation'/exp	117859
#29	'horticultural therapy'/de	222
#28	'aromatherapy'/de	3391
#27	'construction work and architectural phenomena'/de	8958
#26	'environmental planning'/exp	22228
#25	'palliative therapy'/de	125822
#24	'advance care planning'/de	7892
#23	'social support'/exp	135031
#22	'self help'/de	15822
#21	'choir (singing)'/de	142
#20	'singing'/de	4674
#19	'animal assisted therapy'/exp	1746
#18	'therapy animal'/de	39
#17	'occupational therapy'/exp	31424
#16	'spiritual healing'/de	1888
#15	'reflexotherapy'/de	29
#14	'massage'/de	19000
#13	'therapeutic touch'/de	209
#12	'forest bathing'/de	109
#11	'laughter therapy'/exp	270
#10	'psychotherapy'/exp	344638
#9	#1 OR #2 OR #3 OR #4 OR #5 OR #6 OR #7 OR #8	506803
#8	alzheimer*:ti,ab,kw OR amentia*:ti,ab,kw OR 'benson* syndrome*':ti,ab,kw OR 'binswanger* disease*':ti,ab,kw OR 'binswanger* encephalopath*':ti,ab,kw OR dementia*:ti,ab,kw OR 'familial pick* disease*':ti,ab,kw OR 'lewy body	431936

	disease*':ti,ab,kw OR 'mesulam* syndrome*':ti,ab,kw OR 'posterior cortical atroph*':ti,ab,kw OR 'primary progressive aphasia*':ti,ab,kw OR 'subcortical leukoencephalopath*':ti,ab,kw OR 'wilhelmsen lynch disease*':ti,ab,kw	
#7	'primary progressive aphasia'/de	3201
#6	'multiinfarct dementia'/de	15904
#5	'mixed dementia'/de	209
#4	'frontotemporal dementia'/de	19639
#3	'diffuse lewy body disease'/de	13234
#2	'alzheimer disease'/de	276071
#1	'dementia'/de	166156

3. Cochrane Library

Interface: **Wiley** content coverage: - Cochrane Database of Systematic Reviews - April 1996<br>Central Trials - Current content July 1998<br><br>Date of Search: 28 March 2025<br><br>Number of hits: 4,255	Field labels<br><br>• mh = exploded MeSH term<br>• mh ^= non exploded MeSH term<br>• ti,ab,kw = title, abstract and author keywords<br>• NEAR/x = within x words, regardless of order<br>• NEXT = used for truncated phrases<br>• NEXT/x = fixed word order<br>• * = truncation of word for alternate endings<br>• ? = 0-1 letter/number

ID	Search	Hits
#1	[mh ^Dementia]	4108
#2	[mh ^"Alzheimer Disease"]	5516
#3	[mh ^"Lewy Body Disease"]	165
#4	[mh ^"Frontotemporal Dementia"]	128
#5	[mh ^"Mixed Dementias"]	4
#6	[mh ^"Dementia, Vascular"]	377
#7	[mh ^"Aphasia, Primary Progressive"]	106
#8	(alzheimer*:ti,ab,kw OR amentia*:ti,ab,kw OR (benson* NEXT syndrome*):ti,ab,kw OR (binswanger* NEXT disease*):ti,ab,kw OR (binswanger* NEXT encephalopath*):ti,ab,kw OR dementia*:ti,ab,kw OR ("familial" NEXT pick* NEXT disease*):ti,ab,kw OR ("lewy body" NEXT disease*):ti,ab,kw OR (mesulam* NEXT syndrome*):ti,ab,kw OR ("posterior cortical" NEXT atroph*):ti,ab,kw OR ("primary progressive" NEXT aphasia*):ti,ab,kw OR ("subcortical" NEXT leukoencephalopath*):ti,ab,kw OR ("wilhelmsen lynch" NEXT disease*):ti,ab,kw)	26865
#9	#1 OR #2 OR #3 OR #4 OR #5 OR #6 OR #7 OR #8	26866
#10	[mh Psychotherapy]	35935
#11	[mh ^"Laughter Therapy"]	91
#12	[mh ^"Mental Healing"]	39
#13	[mh ^"Forest Therapy"]	0
#14	[mh ^"Therapeutic Touch"]	191
#15	[mh ^Massage]	1626
#16	[mh ^Reflexotherapy]	35
#17	[mh "Sensory Art Therapies"]	3651

#18	[mh "Spiritual Therapies"]	2421
#19	[mh ^"Occupational Therapy"]	1032
#20	[mh ^"Therapy Animals"]	6
#21	[mh ^Singing]	106
#22	[mh ^"Self-Help Groups"]	860
#23	[mh ^"Social Support"]	4292
#24	[mh ^"Advance Care Planning"]	367
#25	[mh ^"Palliative Care"]	2605
#26	[mh "Environment Design"]	189
#27	[mh "Facility Design and Construction"]	277
#28	[mh "Environment, Controlled"]	3847
#29	(aromatherap*:ti,ab,kw OR bibliotherap*:ti,ab,kw OR chromatotherap*:ti,ab,kw OR chromotherap*:ti,ab,kw OR dramatherap*:ti,ab,kw OR ecotherap*:ti,ab,kw OR psychotherap*:ti,ab,kw OR reflexotherap*:ti,ab,kw)	20420
#30	((caregiver:ti,ab,kw OR complementary:ti,ab,kw OR family:ti,ab,kw OR group:ti,ab,kw OR narrative:ti,ab,kw OR occupational:ti,ab,kw OR play:ti,ab,kw) NEXT (intervention*:ti,ab,kw OR program*:ti,ab,kw OR therap*:ti,ab,kw OR treatment*:ti,ab,kw))	27690
#31	(("crisis" NEXT intervention*):ti,ab,kw OR ("self-help" NEXT group*):ti,ab,kw OR ("sensitivity training" NEXT group*):ti,ab,kw)	587
#32	(("acceptance and commitment":ti,ab,kw OR "acoustic stimulation":ti,ab,kw OR "anger management":ti,ab,kw OR ("applied" NEXT behavio?r NEXT analys?s):ti,ab,kw OR aroma:ti,ab,kw OR aversion:ti,ab,kw OR "auditory stimulation":ti,ab,kw OR aversive:ti,ab,kw OR behavior-change:ti,ab,kw OR "client centered":ti,ab,kw OR clown:ti,ab,kw OR colo?r:ti,ab,kw OR compassion*:ti,ab,kw OR "communication training":ti,ab,kw OR ("compensatory" NEXT strateg*):ti,ab,kw OR conditioning:ti,ab,kw OR (conversation* NEXT "coaching"):ti,ab,kw OR coping:ti,ab,kw OR culture-based:ti,ab,kw OR couple:ti,ab,kw OR ("dialectical" NEXT behavio?r*):ti,ab,kw OR dignity:ti,ab,kw OR "directed reverie":ti,ab,kw OR doll?:ti,ab,kw OR drama:ti,ab,kw OR (emotion* NEXT "focused"):ti,ab,kw OR ("encounter" NEXT group*):ti,ab,kw OR forest:ti,ab,kw OR ("free" NEXT association*):ti,ab,kw OR garden*:ti,ab,kw OR gestalt:ti,ab,kw OR grief:ti,ab,kw OR "guided imagery":ti,ab,kw OR ("health" NEXT educat*):ti,ab,kw OR healing:ti,ab,kw OR horticultur*:ti,ab,kw OR laughter:ti,ab,kw OR "life review":ti,ab,kw OR "laying-on-of-hands":ti,ab,kw OR "life story work":ti,ab,kw OR marital:ti,ab,kw OR marriage:ti,ab,kw OR massage:ti,ab,kw OR meditation:ti,ab,kw OR mindfulness:ti,ab,kw OR ("mixed" NEXT realit*):ti,ab,kw OR "multi	70368

	family":ti,ab,kw OR multifamily:ti,ab,kw OR "multiple family":ti,ab,kw OR multisensory:ti,ab,kw OR multi-sensory:ti,ab,kw OR "namaste care":ti,ab,kw OR nature:ti,ab,kw OR nondirective:ti,ab,kw OR "object handling":ti,ab,kw OR ("peer" NEXT group*):ti,ab,kw OR "personal validation":ti,ab,kw OR photo-voice:ti,ab,kw OR "play-based mental health":ti,ab,kw OR psychoanalytic*:ti,ab,kw OR psychodrama:ti,ab,kw OR psycholog*:ti,ab,kw OR psychosocial*:ti,ab,kw OR reablement:ti,ab,kw OR reality:ti,ab,kw OR reflex:ti,ab,kw OR reiki:ti,ab,kw OR relaxation:ti,ab,kw OR reminiscence:ti,ab,kw OR rogerian:ti,ab,kw OR ("role" NEXT playing*):ti,ab,kw OR sandplay:ti,ab,kw OR "sensory stimulation":ti,ab,kw OR shinrin-yoku:ti,ab,kw OR snoezelen:ti,ab,kw OR "social support":ti,ab,kw OR socioenvironment*:ti,ab,kw OR "solution focused brief":ti,ab,kw OR t-group*:ti,ab,kw OR talking:ti,ab,kw OR touch*:ti,ab,kw) NEAR/2 (intervention*:ti,ab,kw OR program*:ti,ab,kw OR therap*:ti,ab,kw OR treatment*:ti,ab,kw))	
#33	("acoustic stimulation":ti,kw OR "anger management":ti,kw OR ("applied" NEXT behavio?r NEXT analys?s):ti,kw OR aroma:ti,kw OR "auditory stimulation":ti,kw OR choir*:ti,kw OR "communication training":ti,kw OR (conversation* NEXT "coaching"):ti,kw OR ("compensatory" NEXT strateg*):ti,kw OR clown?:ti,kw OR "creative writing":ti,kw OR doll?:ti,kw OR drama:ti,kw OR (emotion* NEXT "focused"):ti,kw OR ("encounter" NEXT group*):ti,kw OR ("forest" NEXT bath*):ti,kw OR ("free" NEXT association*):ti,kw OR garden*:ti,kw OR "grief counseling":ti,kw OR "guided imagery":ti,kw OR horticultur*:ti,kw OR laughter:ti,kw OR handicraft*:ti,kw OR "laying-on-of-hands":ti,kw OR "life story work":ti,kw OR massage:ti,kw OR meditation:ti,kw OR mindfulness:ti,kw OR ("mixed" NEXT realit*):ti,kw OR "multisensory stimulation":ti,kw OR museum*:ti,kw OR music:ti,kw OR "namaste care":ti,kw OR ("peer" NEXT group*):ti,kw OR "personal validation":ti,kw OR photo-voice:ti,kw OR poetry:ti,kw OR psychodrama:ti,kw OR reablement:ti,kw OR "reality orientation":ti,kw OR reiki:ti,kw OR ("relaxation" NEXT techni*):ti,kw OR reminiscence:ti,kw OR ("role" NEXT playing*):ti,kw OR sandplay:ti,kw OR "sensory stimulation":ti,kw OR shinrin-yoku:ti,kw OR snoezelen:ti,kw OR singing:ti,kw OR storytelling:ti,kw OR ("systematic" NEXT therap*):ti,kw OR t-group*:ti,kw OR theatre?:ti,kw)	31968
#34	((handicraft*:ti,ab,kw OR poetry:ti,ab,kw OR reading:ti,ab,kw OR singing:ti,ab,kw OR storytelling:ti,ab,kw OR theatre?:ti,ab,kw) NEAR/2 (activit*:ti,ab,kw OR intervention*:ti,ab,kw OR program*:ti,ab,kw OR therap*:ti,ab,kw OR treatment*:ti,ab,kw))	1112
#35	((cognition:ti,ab,kw OR cognitive:ti,ab,kw OR ("cognitive" NEXT behavio*):ti,ab,kw) NEAR/1 (intervention*:ti,ab,kw OR program*:ti,ab,kw OR rehabilitation:ti,ab,kw OR reframing:ti,ab,kw OR remediation:ti,ab,kw OR restructuring:ti,ab,kw OR therap*:ti,ab,kw OR training:ti,ab,kw))	35348
#36	((("health" NEXT educat*):ti,ab,kw OR nonpharmacolog*:ti,ab,kw OR non-pharmacolog*:ti,ab,kw) NEAR/2 (intervention*:ti,ab,kw OR program*:ti,ab,kw OR therap*:ti,ab,kw OR treatment*:ti,ab,kw))	7659

#37	((art:ti,ab,kw OR choir*:ti,ab,kw OR music:ti,ab,kw OR writing:ti,ab,kw) NEAR/2 (activit*:ti,ab,kw OR alzheimer*:ti,ab,kw OR dementia*:ti,ab,kw OR making:ti,ab,kw OR intervention*:ti,ab,kw OR participat*:ti,ab,kw OR program*:ti,ab,kw OR therap*:ti,ab,kw OR treatment*:ti,ab,kw))	10208
#38	(behavio*:ti,ab,kw NEXT (("change" NEXT technique*):ti,ab,kw OR counsel*:ti,ab,kw OR modification*:ti,ab,kw OR therap*:ti,ab,kw OR treatment*:ti,ab,kw OR support:ti,ab,kw))	37223
#39	(museum*:ti,ab,kw NEAR/2 (activit*:ti,ab,kw OR attend*:ti,ab,kw OR alzheimer*:ti,ab,kw OR dementia*:ti,ab,kw OR intervention*:ti,ab,kw OR participat*:ti,ab,kw OR program*:ti,ab,kw OR therap*:ti,ab,kw OR visit:ti,ab,kw))	31
#40	(community:ti,ab,kw NEAR/2 (activit*:ti,ab,kw OR hub?:ti,ab,kw OR initiative*:ti,ab,kw OR intervention*:ti,ab,kw OR program*:ti,ab,kw))	5745
#41	((alzheimer*:ti,ab,kw OR dementia*:ti,ab,kw OR memory:ti,ab,kw) NEAR/2 cafe?:ti,ab,kw)	7
#42	(("social interaction" NEXT intervention*):ti,ab,kw OR ("social" NEXT intervention*):ti,ab,kw)	273
#43	(("social" NEXT activit*):ti,ab,kw NEAR/2 (intervention*:ti,ab,kw OR program*:ti,ab,kw OR therap*:ti,ab,kw OR treatment*:ti,ab,kw))	47
#44	(("meeting" NEXT centre*):ti,ab,kw NEAR/2 program*:ti,ab,kw)	4
#45	((animal*:ti,ab,kw OR "animal assisted":ti,ab,kw OR dog?:ti,ab,kw OR equine*:ti,ab,kw OR pet?:ti,ab,kw OR "pet assisted":ti,ab,kw) NEAR/2 ("emotional support":ti,ab,kw OR intervention*:ti,ab,kw OR therap*:ti,ab,kw))	1272
#46	((assistive:ti,ab,kw OR companion*:ti,ab,kw) NEAR/2 (animal?:ti,ab,kw OR pet?:ti,ab,kw))	44
#47	((animal*:ti,ab,kw OR human*:ti,ab,kw OR pet?:ti,ab,kw OR social:ti,ab,kw) NEAR/2 robot*:ti,ab,kw)	260
#48	(("care planning":ti,ab,kw OR "end of life":ti,ab,kw OR psychoeducation*:ti,ab,kw OR "palliative care":ti,ab,kw) NEAR/2 (intervention*:ti,ab,kw OR program*:ti,ab,kw OR therap*:ti,ab,kw OR treatment*:ti,ab,kw))	3889
#49	((architectural:ti,ab,kw OR environmental*:ti,ab,kw OR facility:ti,ab,kw OR facilities:ti,ab,kw OR garden*:ti,ab,kw OR "human centered":ti,ab,kw OR residential:ti,ab,kw OR universal:ti,ab,kw) NEAR/2 design*:ti,ab,kw)	343
#50	(("built" NEXT environment*):ti,ab,kw OR (environment* NEXT adaptation*):ti,ab,kw)	249
#51	(home*:ti,ab,kw NEAR/2 modification*:ti,ab,kw)	148
#52	#10 OR #11 OR #12 OR #13 OR #14 OR #15 OR #16 OR #17 OR #18 OR #19 OR #20 OR #21 OR #22 OR #23 OR #24 OR #25 OR #26 OR #27 OR #28 OR #29 OR	180050

	#30 OR #31 OR #32 OR #33 OR #34 OR #35 OR #36 OR #37 OR #38 OR #39 OR #40 OR #41 OR #42 OR #43 OR #44 OR #45 OR #46 OR #47 OR #48 OR #49 OR #50 OR #51	
#53	[mh ^"User-Centered Design"]	16
#54	[mh ^"Program Evaluation"]	7573
#55	[mh ^"Evaluation Studies as Topic"]	4445
#56	[mh ^"Evaluation Study"]	1
#57	[mh ^"Feasibility Studies"]	11154
#58	[mh ^"Implementation Science"]	117
#59	[mh ^"Pilot Projects"]	30151
#60	[mh ^"Comparative Effectiveness Research"]	498
#61	[mh "Costs and Cost Analysis"]	16363
#62	[mh "Clinical Trial"]	42
#63	(acceptability:ti,ab,kw OR adapt*:ti,ab,kw OR adherence:ti,ab,kw OR adoption*:ti,ab,kw OR advantage*:ti,ab,kw OR appropriateness:ti,ab,kw OR barrier?:ti,ab,kw OR ("business" NEXT model*):ti,ab,kw OR cost?:ti,ab,kw OR create:ti,ab,kw OR customiz*:ti,ab,kw OR deliver:ti,ab,kw OR design*:ti,ab,kw OR develop*:ti,ab,kw OR document*:ti,ab,kw OR effect?:ti,ab,kw OR effectiveness:ti,ab,kw OR efficacy:ti,ab,kw OR evaluat*:ti,ab,kw OR exploratory:ti,ab,kw OR facilitator*:ti,ab,kw OR feasibility:ti,ab,kw OR fidelity:ti,ab,kw OR impact*:ti,ab,kw OR implement*:ti,ab,kw OR (mechanism* NEXT "of change"):ti,ab,kw OR penetration:ti,ab,kw OR pilot:ti,ab,kw OR plan:ti,ab,kw OR produce:ti,ab,kw OR production*:ti,ab,kw OR proof-of-concept:ti,ab,kw OR quality:ti,ab,kw OR refine:ti,ab,kw OR sustainability:ti,ab,kw OR testing:ti,ab,kw OR theory:ti,ab,kw OR theoretical:ti,ab,kw OR theories:ti,ab,kw OR trial?:ti,ab,kw OR trialability:ti,ab,kw OR uncertainty:ti,ab,kw OR uptake*:ti,ab,kw OR usability:ti,ab,kw OR user-centered:ti,ab,kw)	1953307
#64	#53 OR #54 OR #55 OR #56 OR #57 OR #58 OR #59 OR #60 OR #61 OR #62 OR #63	1953308
#65	#9 AND #52 AND #64 with Publication Year from 2015 to 2025, in Trials & Cochrane Reviews	4299
#66	#9 AND #52 AND #64 with Publication Year from 2015 to 2025, in Trials & Cochrane Reviews, Limit to english	4255

4. Web of Science Core Collection

Interface: **Clarivate Analytics**<br><br>Editions and content coverage years= A&HCI - 1975 , ESCI - 2019 , SCI-EXPANDED - 1945 , SSCI - 1945<br><br>Date of Search: 28 March 2025<br><br>Number of hits: 11,016	Field labels<br><br>• TS/Topic = title, abstract, author keywords and Keywords Plus<br>• TI= title<br>• AB = abstract<br>• AK = author keywords<br>• NEAR/x = within x words, regardless of order<br>• * = truncation of word for alternate endings<br>• $ = 0-1 letter/number<br>• ? = 1 letter/number<br><br>Note: the *Exact search*-function was used for all the searches

#	**Search Query**	**Results**
1	TS=(acceptability OR adapt* OR adherence OR adoption* OR advantage* OR appropriateness OR barrier$ OR "business model*" OR cost$ OR create OR customiz* OR deliver OR design* OR develop* OR document* OR effect$ OR effectiveness OR efficacy OR evaluat* OR exploratory OR facilitator* OR feasibility OR fidelity OR impact* OR implement* OR "mechanism* of change" OR penetration OR pilot OR plan OR produce OR production* OR proof-of-concept OR quality OR refine OR sustainability OR testing OR theory OR theoretical OR theories OR trial$ OR trialability OR uncertainty OR uptake* OR usability OR user-centered)	39515968
2	TS=(home* NEAR/1 modification*)	827
3	TS=("built environment*" OR "environment* adaptation*")	35155
4	TS=((architectural OR environmental* OR facility OR facilities OR garden* OR "human centered" OR residential OR universal) NEAR/1 design*)	28394
5	TS=(("care planning" OR "end of life" OR psychoeducation* OR "palliative care") NEAR/1 (intervention* OR program* OR therap* OR treatment*))	10442
6	TS=((animal* OR human* OR pet$ OR social) NEAR/1 robot*)	20783
7	TS=((assistive OR companion*) NEAR/1 (animal$ OR pet$))	7993
8	TS=((animal* OR "animal assisted" OR dog$ OR equine* OR pet$ OR "pet assisted") NEAR/1 ("emotional support" OR intervention* OR therap*))	8356
9	TS=("meeting centre*" NEAR/1 program*)	21
10	TS=("social activit*" NEAR/1 (intervention* OR program* OR therap* OR treatment*))	117
11	TS=("social interaction intervention*" OR "social intervention*")	3374
12	TS=((alzheimer* OR dementia* OR memory) NEAR/1 cafe$)	44
13	TS=(community NEAR/1 (activit* OR hub$ OR initiative* OR intervention* OR program*))	36382
14	TS=(museum* NEAR/1 (activit* OR attend* OR alzheimer* OR dementia* OR intervention* OR participat* OR program* OR therap* OR visit))	1338
15	TS=(behavio* NEAR/0 ("change technique*" OR counsel* OR modification* OR therap* OR treatment* OR support))	90970
16	TS=((art OR choir* OR music OR writing) NEAR/1 (activit* OR alzheimer* OR dementia* OR making OR intervention* OR participat* OR program* OR therap* OR treatment*))	52264
17	TS=(("health educat*" OR nonpharmacolog* OR non-pharmacolog*) NEAR/1 (intervention* OR program* OR therap* OR treatment*))	23949

18	TS=((cognition OR cognitive OR "cognitive behavio*") NEAR/1 (intervention* OR program* OR rehabilitation OR reframing OR remediation OR restructuring OR therap* OR training))	84674
19	TS=((handicraft* OR poetry OR reading OR singing OR storytelling OR theatre$) NEAR/1 (activit* OR intervention* OR program* OR therap* OR treatment*))	8491
20	TI=("acoustic stimulation" OR "anger management" OR "applied behavio$r analys?s" OR aroma OR "auditory stimulation" OR choir* OR "communication training" OR "conversation* coaching" OR "compensatory strateg*" OR clown$ OR "creative writing" OR doll$ OR drama OR "emotion* focused" OR "encounter group*" OR "forest bath*" OR "free association*" OR garden* OR "grief counseling" OR "guided imagery" OR horticultur* OR laughter OR handicraft* OR laying-on-of-hands OR "life story work" OR massage OR meditation OR mindfulness OR "mixed realit*" OR "multisensory stimulation" OR museum* OR music OR "namaste care" OR "peer group*" OR "personal validation" OR photo-voice OR poetry OR psychodrama OR reablement OR "reality orientation" OR reiki OR "relaxation techni*" OR reminiscence OR "role playing*" OR sandplay OR "sensory stimulation" OR shinrin-yoku OR snoezelen OR singing OR storytelling OR "systematic therap*" OR t-group* OR theatre$) OR AK=("acoustic stimulation" OR "anger management" OR "applied behavio$r analys?s" OR aroma OR "auditory stimulation" OR choir* OR "communication training" OR "conversation* coaching" OR "compensatory strateg*" OR clown$ OR "creative writing" OR doll$ OR drama OR "emotion* focused" OR "encounter group*" OR "forest bath*" OR "free association*" OR garden* OR "grief counseling" OR "guided imagery" OR horticultur* OR laughter OR handicraft* OR laying-on-of-hands OR "life story work" OR massage OR meditation OR mindfulness OR "mixed realit*" OR "multisensory stimulation" OR museum* OR music OR "namaste care" OR "peer group*" OR "personal validation" OR photo-voice OR poetry OR psychodrama OR reablement OR "reality orientation" OR reiki OR "relaxation techni*" OR reminiscence OR "role playing*" OR sandplay OR "sensory stimulation" OR shinrin-yoku OR snoezelen OR singing OR storytelling OR "systematic therap*" OR t-group* OR theatre$) OR KP=("acoustic stimulation" OR "anger management" OR "applied behavio$r analys?s" OR aroma OR "auditory stimulation" OR choir* OR "communication training" OR "conversation* coaching" OR "compensatory strateg*" OR clown$ OR "creative writing" OR doll$ OR drama OR "emotion* focused" OR "encounter group*" OR "forest bath*" OR "free association*" OR garden* OR "grief counseling" OR "guided imagery" OR horticultur* OR laughter OR handicraft* OR laying-on-of-hands OR "life story work" OR massage OR meditation OR mindfulness OR "mixed realit*" OR "multisensory stimulation" OR museum* OR music OR "namaste care" OR "peer group*" OR "personal validation" OR photo-voice OR poetry OR psychodrama OR reablement OR "reality orientation" OR reiki OR "relaxation techni*" OR reminiscence OR "role playing*" OR sandplay OR "sensory stimulation" OR shinrin-yoku OR snoezelen OR singing OR storytelling OR "systematic therap*" OR t-group* OR theatre$)	486040
21	TS=(("acceptance and commitment" OR "acoustic stimulation" OR "anger management" OR "applied behavio$r analys?s" OR aroma OR aversion OR "auditory stimulation" OR aversive OR behavior-change OR "client centered" OR clown OR colo$r OR compassion* OR "communication training" OR "compensatory strateg*" OR conditioning OR "conversation* coaching" OR coping OR culture-based OR couple OR "dialectical behavio$r*" OR dignity OR "directed reverie" OR doll$ OR drama OR "emotion* focused" OR "encounter group*" OR forest OR "free association*" OR garden* OR gestalt OR grief OR "guided imagery" OR "health educat*" OR healing OR horticultur* OR laughter OR "life review" OR laying-on-of-hands OR "life story work" OR marital OR	124969

	marriage OR massage OR meditation OR mindfulness OR "mixed realit*" OR "multi family" OR multifamily OR "multiple family" OR multisensory OR multi-sensory OR "namaste care" OR nature OR nondirective OR "object handling" OR "peer group*" OR "personal validation" OR photo-voice OR "play-based mental health" OR psychoanalytic* OR psychodrama OR psycholog* OR psychosocial* OR psycho-social* OR reablement OR reality OR reflex OR reiki OR relaxation OR reminiscence OR rogerian OR "role playing*" OR sandplay OR "sensory stimulation" OR shinrin-yoku OR snoezelen OR "social support" OR socioenvironment* OR socio-environment* OR "solution focused brief" OR t-group* OR talking OR touch*) NEAR/1 (intervention* OR program* OR therap* OR treatment*))	
22	TS=("crisis intervention*" OR "self-help group*" OR "sensitivity training group*")	5838
23	TS=((caregiver OR complementary OR family OR group OR narrative OR occupational OR play) NEAR/0 (intervention* OR program* OR therap* OR treatment*))	204495
24	TS=(aromatherap* OR bibliotherap* OR chromatotherap* OR chromotherap* OR dramatherap* OR ecotherap* OR psychotherap* OR reflexotherap*)	108786
25	#2 OR #3 OR #4 OR #5 OR #6 OR #7 OR #8 OR #9 OR #10 OR #11 OR #12 OR #13 OR #14 OR #15 OR #16 OR #17 OR #18 OR #19 OR #20 OR #21 OR #22 OR #23 OR #24	1145300
26	TS=(alzheimer* OR amentia* OR "benson* syndrome*" OR "binswanger* disease*" OR "binswanger* encephalopath*" OR dementia* OR "familial pick* disease*" OR "lewy body disease*" OR "mesulam* syndrome*" OR "posterior cortical atroph*" OR "primary progressive aphasia*" OR "subcortical leukoencephalopath*" OR "wilhelmsen lynch disease*")	454359
27	(#1 AND #25 AND #26) AND ((PY==("2025" OR "2024" OR "2023" OR "2022" OR "2021" OR "2020" OR "2019" OR "2018" OR "2017" OR "2016" OR "2015") AND LA==("ENGLISH")) NOT (DT==("MEETING ABSTRACT" OR "EDITORIAL MATERIAL" OR "LETTER")))	11016

5. PsycINFO

Interface: **EBSCOhost** - content coverage from 1806<br><br>Date of Search: 28 March 2025<br><br>Number of hits: 5,365	Field labels<br><br>• DE = subject heading<br>• TI = title<br>• AB = abstract<br>• KW = author keywords<br>• Nx = within x words, regardless of order<br>• * = truncation of word for alternate endings<br>• # = 0-1 letter/number<br>• ? = 1 letter/number<br><br>Note: sometimes “quotation marks” are needed for single search terms to avoid automatic term mapping (lemmatization)

#	Query	Limiters/Expanders	Results
S41	S3 AND S33 AND S38	Limiters - Publication Year: 2015-2025<br>Expanders - Apply equivalent subjects<br>Narrow by Language: - english	5,365

		Search modes - Find all my search terms	
S40	S3 AND S33 AND S38	Limiters - Publication Year: 2015-2025<br>Expanders - Apply equivalent subjects<br>Search modes - Find all my search terms	5,595
S39	S3 AND S33 AND S38	Expanders - Apply equivalent subjects<br>Search modes - Find all my search terms	10,454
S38	S34 OR S35 OR S36 OR S37	Expanders - Apply equivalent subjects<br>Search modes - Find all my search terms	4,129,548
S37	TI ((acceptability OR adapt* OR adherence OR adoption* OR advantage* OR appropriateness OR barrier# OR "business model*" OR cost# OR create OR customis* OR customiz* OR deliver OR design* OR develop* OR document* OR effect# OR effectiveness OR efficacy OR evaluat* OR exploratory OR facilitator* OR feasibility OR fidelity OR impact* OR implement* OR "mechanism* of change" OR penetration OR pilot OR plan OR produce OR production* OR proof-of-concept OR quality OR refine OR sustainability OR testing OR theory OR theoretical OR theories OR trial# OR trialability OR uncertainty OR uptake* OR usability OR user-centered)) OR AB ((acceptability OR adapt* OR adherence OR adoption* OR advantage* OR appropriateness OR barrier# OR "business model*" OR cost# OR create OR customis* OR customiz* OR deliver OR design* OR develop* OR document* OR effect# OR effectiveness OR efficacy OR evaluat* OR exploratory OR facilitator* OR feasibility OR fidelity OR impact* OR implement* OR "mechanism* of change" OR penetration OR pilot OR plan OR produce OR production* OR proof-of-concept OR quality OR refine OR sustainability OR testing OR theory OR theoretical OR theories OR trial# OR trialability OR uncertainty OR uptake* OR usability OR user-centered)) OR KW ((acceptability OR adapt* OR adherence OR adoption* OR advantage* OR appropriateness OR barrier# OR "business model*" OR cost# OR create OR customis* OR customiz* OR	Expanders - Apply equivalent subjects<br>Search modes - Find all my search terms	4,125,002

	deliver OR design* OR develop* OR document* OR effect# OR effectiveness OR efficacy OR evaluat* OR exploratory OR facilitator* OR feasibility OR fidelity OR impact* OR implement* OR "mechanism* of change" OR penetration OR pilot OR plan OR produce OR production* OR proof-of-concept OR quality OR refine OR sustainability OR testing OR theory OR theoretical OR theories OR trial# OR trialability OR uncertainty OR uptake* OR usability OR user-centered))		
S36	(DE "Clinical Trials" OR DE "Randomized Controlled Trials" OR DE "Randomized Clinical Trials" OR DE "Treatment Effectiveness Evaluation")	Expanders - Apply equivalent subjects Search modes - Find all my search terms	43,934
S35	DE "Costs and Cost Analysis" OR DE "Health Care Costs"	Expanders - Apply equivalent subjects Search modes - Find all my search terms	33,145
S34	(DE "Program Evaluation" OR DE "Program Development")	Expanders - Apply equivalent subjects Search modes - Find all my search terms	36,334
S33	S4 OR S5 OR S6 OR S7 OR S8 OR S9 OR S10 OR S11 OR S12 OR S13 OR S14 OR S15 OR S16 OR S17 OR S18 OR S19 OR S20 OR S21 OR S22 OR S23 OR S24 OR S25 OR S26 OR S27 OR S28 OR S29 OR S30 OR S31 OR S32	Expanders - Apply equivalent subjects Search modes - Find all my search terms	717,124
S32	TI (home* N2 modification*) OR AB (home* N2 modification*) OR KW (home* N2 modification*)	Expanders - Apply equivalent subjects Search modes - Find all my search terms	309
S31	TI (("built environment*" OR "environment* adaptation*")) OR AB (("built environment*" OR "environment* adaptation*")) OR KW (("built environment*" OR "environment* adaptation*"))	Expanders - Apply equivalent subjects Search modes - Find all my search terms	3,423
S30	TI (((architectural OR environmental* OR facility OR facilities OR garden* OR "human centered" OR residential OR universal) N2 design*)) OR AB (((architectural OR environmental* OR facility OR facilities OR garden* OR "human centered" OR residential OR universal) N2 design*)) OR KW (	Expanders - Apply equivalent subjects Search modes - Find all my search terms	4,195

	((architectural OR environmental* OR facility OR facilities OR garden* OR "human centered" OR residential OR universal) N2 design*))		
S29	TI ((("care planning" OR "end of life" OR psychoeducation* OR psycho-education* OR "palliative care") N2 (intervention* OR program* OR therap* OR treatment*))) OR AB ((("care planning" OR "end of life" OR psychoeducation* OR psycho-education* OR "palliative care") N2 (intervention* OR program* OR therap* OR treatment*))) OR KW ((("care planning" OR "end of life" OR psychoeducation* OR psycho-education* OR "palliative care") N2 (intervention* OR program* OR therap* OR treatment*)))	Expanders - Apply equivalent subjects<br>Search modes - Find all my search terms	8,306
S28	TI (((animal* OR human* OR pet# OR social) N2 robot*)) OR AB (((animal* OR human* OR pet# OR social) N2 robot*)) OR KW (((animal* OR human* OR pet# OR social) N2 robot*))	Expanders - Apply equivalent subjects<br>Search modes - Find all my search terms	4,157
S27	TI (((assistive OR companion*) N2 (animal# OR pet#))) OR AB (((assistive OR companion*) N2 (animal# OR pet#))) OR KW (((assistive OR companion*) N2 (animal# OR pet#)))	Expanders - Apply equivalent subjects<br>Search modes - Find all my search terms	1,336
S26	TI (((animal* OR "animal assisted" OR dog# OR equine* OR pet# OR "pet assisted") N2 ("emotional support" OR intervention* OR therap*))) OR AB (((animal* OR "animal assisted" OR dog# OR equine* OR pet# OR "pet assisted") N2 ("emotional support" OR intervention* OR therap*))) OR KW (((animal* OR "animal assisted" OR dog# OR equine* OR pet# OR "pet assisted") N2 ("emotional support" OR intervention* OR therap*)))	Expanders - Apply equivalent subjects<br>Search modes - Find all my search terms	2,393
S25	TI ("meeting centre*" N2 program*) OR AB ("meeting centre*" N2 program*) OR KW ("meeting centre*" N2 program*)	Expanders - Apply equivalent subjects<br>Search modes - Find all my search terms	17
S24	TI (("social activit*" N2 (intervention* OR program* OR therap* OR treatment*))) OR AB (("social activit*" N2 (intervention* OR program* OR therap* OR treatment*))) OR KW (("social activit*" N2 (intervention* OR program* OR therap* OR treatment*)))	Expanders - Apply equivalent subjects<br>Search modes - Find all my search terms	103

S23	TI (("social interaction intervention*" OR "social intervention*")) OR AB (("social interaction intervention*" OR "social intervention*")) OR KW (("social interaction intervention*" OR "social intervention*"))	Expanders - Apply equivalent subjects<br>Search modes - Find all my search terms	1,703
S22	TI (((alzheimer* OR dementia* OR memory) N2 cafe#)) OR AB (((alzheimer* OR dementia* OR memory) N2 cafe#)) OR KW (((alzheimer* OR dementia* OR memory) N2 cafe#))	Expanders - Apply equivalent subjects<br>Search modes - Find all my search terms	25
S21	TI ((community N2 (activit* OR hub# OR initiative* OR intervention* OR program*))) OR AB ((community N2 (activit* OR hub# OR initiative* OR intervention* OR program*))) OR KW ((community N2 (activit* OR hub# OR initiative* OR intervention* OR program*)))	Expanders - Apply equivalent subjects<br>Search modes - Find all my search terms	27,115
S20	TI ((museum* N2 (activit* OR attend* OR alzheimer* OR dementia* OR intervention* OR participat* OR program* OR therap* OR visit))) OR AB ((museum* N2 (activit* OR attend* OR alzheimer* OR dementia* OR intervention* OR participat* OR program* OR therap* OR visit))) OR KW ((museum* N2 (activit* OR attend* OR alzheimer* OR dementia* OR intervention* OR participat* OR program* OR therap* OR visit)))	Expanders - Apply equivalent subjects<br>Search modes - Find all my search terms	485
S19	TI ((behavio* W1 ("change technique*" OR counsel* OR modification* OR therap* OR treatment* OR support))) OR AB ((behavio* W1 ("change technique*" OR counsel* OR modification* OR therap* OR treatment* OR support))) OR KW ((behavio* W1 ("change technique*" OR counsel* OR modification* OR therap* OR treatment* OR support)))	Expanders - Apply equivalent subjects<br>Search modes - Find all my search terms	71,731
S18	TI (((art OR choir* OR music OR writing) N2 (activit* OR alzheimer* OR dementia* OR making OR intervention* OR participat* OR program* OR therap* OR treatment*))) OR AB (((art OR choir* OR music OR writing) N2 (activit* OR alzheimer* OR dementia* OR making OR intervention* OR participat* OR program* OR therap* OR treatment*))) OR KW (((art OR choir* OR music OR writing) N2 (activit* OR alzheimer* OR dementia* OR making OR intervention* OR participat* OR program* OR therap* OR treatment*)))	Expanders - Apply equivalent subjects<br>Search modes - Find all my search terms	27,251

S17	TI ((("health educat*" OR nonpharmacolog* OR non-pharmacolog*) N2 (intervention* OR program* OR therap* OR treatment*))) OR AB ((("health educat*" OR nonpharmacolog* OR non-pharmacolog*) N2 (intervention* OR program* OR therap* OR treatment*))) OR KW ((("health educat*" OR nonpharmacolog* OR non-pharmacolog*) N2 (intervention* OR program* OR therap* OR treatment*)))	Expanders - Apply equivalent subjects<br>Search modes - Find all my search terms	7,847
S16	TI (((cognition OR cognitive OR "cognitive behavio*") N1 (intervention* OR program* OR rehabilitation OR reframing OR remediation OR restructuring OR therap* OR training))) OR AB (((cognition OR cognitive OR "cognitive behavio*") N1 (intervention* OR program* OR rehabilitation OR reframing OR remediation OR restructuring OR therap* OR training))) OR KW (((cognition OR cognitive OR "cognitive behavio*") N1 (intervention* OR program* OR rehabilitation OR reframing OR remediation OR restructuring OR therap* OR training)))	Expanders - Apply equivalent subjects<br>Search modes - Find all my search terms	60,656
S15	TI (((handicraft* OR poetry OR reading OR singing OR storytelling OR theatre#) N2 (activit* OR intervention* OR program* OR therap* OR treatment*))) OR AB (((handicraft* OR poetry OR reading OR singing OR storytelling OR theatre#) N2 (activit* OR intervention* OR program* OR therap* OR treatment*))) OR KW (((handicraft* OR poetry OR reading OR singing OR storytelling OR theatre#) N2 (activit* OR intervention* OR program* OR therap* OR treatment*)))	Expanders - Apply equivalent subjects<br>Search modes - Find all my search terms	10,848
S14	TI (("acoustic stimulation" OR "anger management" OR "applied behavio#r analys?s" OR aroma OR "auditory stimulation" OR choir* OR "communication training" OR "conversation* coaching" OR "compensatory strateg*" OR clown# OR "creative writing" OR doll# OR drama OR "emotion* focused" OR "encounter group*" OR "forest bath*" OR "free association*" OR garden* OR "grief counseling" OR "guided imagery" OR horticultur* OR laughter OR handicraft* OR laying-on-of-hands OR "life story work" OR massage OR meditation OR mindfulness OR "mixed realit*" OR "multisensory stimulation" OR museum* OR music OR "namaste care" OR "peer group*" OR "personal validation" OR photo-voice OR poetry OR psychodrama OR reablement OR "reality orientation" OR reiki OR "relaxation techni*" OR	Expanders - Apply equivalent subjects<br>Search modes - Find all my search terms	84,164

	reminiscence OR "role playing*" OR sandplay OR "sensory stimulation" OR shinrin-yoku OR snoezelen OR singing OR storytelling OR "systematic therap*" OR t-group* OR theatre#)) OR KW (("acoustic stimulation" OR "anger management" OR "applied behavio#r analys?s" OR aroma OR "auditory stimulation" OR choir* OR "communication training" OR "conversation* coaching" OR "compensatory strateg*" OR clown# OR "creative writing" OR doll# OR drama OR "emotion* focused" OR "encounter group*" OR "forest bath*" OR "free association*" OR garden* OR "grief counseling" OR "guided imagery" OR horticultur* OR laughter OR handicraft* OR laying-on-of-hands OR "life story work" OR massage OR meditation OR mindfulness OR "mixed realit*" OR "multisensory stimulation" OR museum* OR music OR "namaste care" OR "peer group*" OR "personal validation" OR photo-voice OR poetry OR psychodrama OR reablement OR "reality orientation" OR reiki OR "relaxation techni*" OR reminiscence OR "role playing*" OR sandplay OR "sensory stimulation" OR shinrin-yoku OR snoezelen OR singing OR storytelling OR "systematic therap*" OR t-group* OR theatre#))		
S13	TI ((("acceptance and commitment" OR "acoustic stimulation" OR "anger management" OR "applied behavio#r analys?s" OR aroma OR aversion OR "auditory stimulation" OR aversive OR behavior-change OR "client centered" OR clown OR colo#r OR compassion* OR "communication training" OR "compensatory strateg*" OR conditioning OR "conversation* coaching" OR coping OR culture-based OR couple OR "dialectical behavio#r*" OR dignity OR "directed reverie" OR doll# OR drama OR "emotion* focused" OR "encounter group*" OR forest OR "free association*" OR garden* OR gestalt OR grief OR "guided imagery" OR "health educat*" OR healing OR horticultur* OR laughter OR "life review" OR laying-on-of-hands OR "life story work" OR marital OR marriage OR massage OR meditation OR mindfulness OR "mixed realit*" OR "multi family" OR multifamily OR "multiple family" OR multisensory OR multi-sensory OR "namaste care" OR nature OR nondirective OR "object handling" OR "peer group*" OR "personal validation" OR photo-voice OR "play-based mental health" OR psychoanalytic* OR psychodrama OR psycholog* OR psychosocial* OR psycho-social* OR reablement OR reality OR reflex OR reiki OR relaxation OR reminiscence OR rogerian OR "role	Expanders - Apply equivalent subjects<br>Search modes - Find all my search terms	130,885

	playing*" OR sandplay OR "sensory stimulation" OR shinrin-yoku OR snoezelen OR "social support" OR socioenvironment* OR socio-environment* OR "solution focused brief" OR t-group* OR talking OR touch*) N2 (intervention* OR program* OR therap* OR treatment*))) OR AB ((("acceptance and commitment" OR "acoustic stimulation" OR "anger management" OR "applied behavio#r analys?s" OR aroma OR aversion OR "auditory stimulation" OR aversive OR behavior-change OR "client centered" OR clown OR colo#r OR compassion* OR "communication training" OR "compensatory strateg*" OR conditioning OR "conversation* coaching" OR coping OR culture-based OR couple OR "dialectical behavio#r*" OR dignity OR "directed reverie" OR doll# OR drama OR "emotion* focused" OR "encounter group*" OR forest OR "free association*" OR garden* OR gestalt OR grief OR "guided imagery" OR "health educat*" OR healing OR horticultur* OR laughter OR "life review" OR laying-on-of-hands OR "life story work" OR marital OR marriage OR massage OR meditation OR mindfulness OR "mixed realit*" OR "multi family" OR multifamily OR "multiple family" OR multisensory OR multi-sensory OR "namaste care" OR nature OR nondirective OR "object handling" OR "peer group*" OR "personal validation" OR photo-voice OR "play-based mental health" OR psychoanalytic* OR psychodrama OR psycholog* OR psychosocial* OR psycho-social* OR reablement OR reality OR reflex OR reiki OR relaxation OR reminiscence OR rogerian OR "role playing*" OR sandplay OR "sensory stimulation" OR shinrin-yoku OR snoezelen OR "social support" OR socioenvironment* OR socio-environment* OR "solution focused brief" OR t-group* OR talking OR touch*) N2 (intervention* OR program* OR therap* OR treatment*))) OR KW ((("acceptance and commitment" OR "acoustic stimulation" OR "anger management" OR "applied behavio#r analys?s" OR aroma OR aversion OR "auditory stimulation" OR aversive OR behavior-change OR "client centered" OR clown OR colo#r OR compassion* OR "communication training" OR "compensatory strateg*" OR conditioning OR "conversation* coaching" OR coping OR culture-based OR couple OR "dialectical behavio#r*" OR dignity OR "directed reverie" OR doll# OR drama OR "emotion* focused" OR "encounter group*" OR forest OR "free association*" OR garden* OR gestalt OR grief OR "guided imagery" OR "health educat*" OR healing OR horticultur* OR laughter OR "life review" OR laying-on-of-hands OR "life story work" OR marital OR		

	marriage OR massage OR meditation OR mindfulness OR "mixed realit*" OR "multi family" OR multifamily OR "multiple family" OR multisensory OR multi-sensory OR "namaste care" OR nature OR nondirective OR "object handling" OR "peer group*" OR "personal validation" OR photo-voice OR "play-based mental health" OR psychoanalytic* OR psychodrama OR psycholog* OR psychosocial* OR psycho-social* OR reablement OR reality OR reflex OR reiki OR relaxation OR reminiscence OR rogerian OR "role playing*" OR sandplay OR "sensory stimulation" OR shinrin-yoku OR snoezelen OR "social support" OR socioenvironment* OR socio-environment* OR "solution focused brief" OR t-group* OR talking OR touch*) N2 (intervention* OR program* OR therap* OR treatment*)))		
S12	TI (("crisis intervention*" OR "self-help group*" OR "sensitivity training group*")) OR AB (("crisis intervention*" OR "self-help group*" OR "sensitivity training group*")) OR KW (("crisis intervention*" OR "self-help group*" OR "sensitivity training group*"))	Expanders - Apply equivalent subjects<br>Search modes - Find all my search terms	6,739
S11	TI (((caregiver OR complementary OR family OR group OR narrative OR occupational OR play) W1 (intervention* OR program* OR therap* OR treatment*))) OR AB (((caregiver OR complementary OR family OR group OR narrative OR occupational OR play) W1 (intervention* OR program* OR therap* OR treatment*))) OR KW (((caregiver OR complementary OR family OR group OR narrative OR occupational OR play) W1 (intervention* OR program* OR therap* OR treatment*)))	Expanders - Apply equivalent subjects<br>Search modes - Find all my search terms	93,706
S10	TI ((aromatherap* OR bibliotherap* OR chromatotherap* OR chromotherap* OR dramatherap* OR ecotherap* OR psychotherap* OR reflexotherap*)) OR AB ((aromatherap* OR bibliotherap* OR chromatotherap* OR chromotherap* OR dramatherap* OR ecotherap* OR psychotherap* OR reflexotherap*)) OR KW ((aromatherap* OR bibliotherap* OR chromatotherap* OR chromotherap* OR dramatherap* OR ecotherap* OR psychotherap* OR reflexotherap*))	Expanders - Apply equivalent subjects<br>Search modes - Find all my search terms	139,383
S9	(DE "Aromatherapy" OR DE "Massage" OR DE "Occupational Therapy" OR DE "Service Animals" OR DE "Singing" OR DE "Self-Help Techniques" OR DE "Support Groups" OR DE "Social Support" OR DE "Palliative Care" OR DE "Environmental Adaptation"	Expanders - Apply equivalent subjects<br>Search modes - Find all my search terms	113,991

	OR DE "Interior Design" OR DE "Furniture" OR DE "Built Environment")		
S8	(DE "Milieu Therapy" OR DE "Bibliotherapy" OR DE "Poetry Therapy" OR DE "Mindfulness-Based Interventions" OR DE "Mindfulness-Based Stress Reduction" OR DE "Psychoeducation" OR DE "Psychosocial Interventions" OR DE "Cognitive Stimulation Therapy")	Expanders - Apply equivalent subjects<br>Search modes - Find all my search terms	13,574
S7	DE "Creative Arts Therapy" OR DE "Art Therapy" OR DE "Dance Therapy" OR DE "Music Therapy"	Expanders - Apply equivalent subjects<br>Search modes - Find all my search terms	14,896
S6	(DE "Nature-Based Interventions" OR DE "Horticulture Therapy" OR DE "Animal Assisted Therapy" OR DE "Equine Assisted Therapy")	Expanders - Apply equivalent subjects<br>Search modes - Find all my search terms	1,947
S5	(DE "Cognitive Behavior Therapy" OR DE "Acceptance and Commitment Therapy" OR DE "Cognitive Analytic Therapy" OR DE "Cognitive Processing Therapy" OR DE "Cognitive Therapy" OR DE "Mindfulness-Based Cognitive Therapy" OR DE "Trauma-Focused Cognitive Behavior Therapy" OR DE "Cognitive Techniques" OR DE "Mindfulness" OR DE "Self-Instructional Training" OR DE "Cognitive Restructuring" OR DE "Metacognitive Therapy" OR DE "Behavior Therapy" OR DE "Applied Behavior Analysis" OR DE "Dialectical Behavior Therapy" OR DE "Implosive Therapy" OR DE "Reciprocal Inhibition Therapy" OR DE "Aversion Therapy" OR DE "Covert Sensitization" OR DE "Behavior Modification" OR DE "Relaxation Therapy" OR DE "Breathing Techniques" OR DE "Progressive Relaxation Therapy" OR DE "Meditation" OR DE "Mindfulness Meditation")	Expanders - Apply equivalent subjects<br>Search modes - Find all my search terms	110,175
S4	(DE "Psychotherapy" OR DE "Adlerian Psychotherapy" OR DE "Affirmative Therapy" OR DE "Analytical Psychotherapy" OR DE "Autogenic Training" OR DE "Brief Relational Therapy" OR DE "Client Centered Therapy" OR DE "Compassion Focused Therapy" OR DE "Couples Therapy" OR DE "Eclectic Psychotherapy" OR DE "Educational Therapy" OR DE "Emotion Focused Therapy" OR DE "Existential Therapy" OR DE "Experiential Psychotherapy" OR DE "Expressive Psychotherapy" OR DE "Eye Movement	Expanders - Apply equivalent subjects<br>Search modes - Find all my search terms	246,951

	Desensitization Therapy" OR DE "Feminist Therapy" OR DE "Geriatric Psychotherapy" OR DE "Guided Imagery" OR DE "Individual Psychotherapy" OR DE "Interpersonal Psychotherapy" OR DE "Logotherapy" OR DE "Metacognitive Therapy" OR DE "Narrative Therapy" OR DE "Network Therapy" OR DE "Personal Therapy" OR DE "Persuasion Therapy" OR DE "Positive Psychology Therapy" OR DE "Primal Therapy" OR DE "Psychodrama" OR DE "Psychodynamic Psychotherapy" OR DE "Rational Emotive Behavior Therapy" OR DE "Reality Therapy" OR DE "Relationship Therapy" OR DE "Solution Focused Therapy" OR DE "Spiritually Oriented Therapy" OR DE "Supportive Psychotherapy" OR DE "Transactional Analysis" OR DE "Brief Psychotherapy" OR DE "Cognitive Analytic Therapy" OR DE "Trauma-Focused Cognitive Behavior Therapy" OR DE "Strategic Therapy" OR DE "Strategic Family Therapy" OR DE "Play Therapy" OR DE "Drama Therapy" OR DE "Gestalt Therapy" OR DE "Empty Chair Technique" OR DE "Group Psychotherapy" OR DE "Therapeutic Community" OR DE "Encounter Group Therapy" OR DE "Marathon Group Therapy" OR DE "Humanistic Psychotherapy" OR DE "Hypnotherapy" OR DE "Age Regression (Hypnotic)" OR DE "Ericksonian Psychotherapy" OR DE "Posthypnotic Suggestions" OR DE "Integrative Psychotherapy" OR DE "Schema Therapy" OR DE "Psychoanalysis" OR DE "Dream Analysis" OR DE "Self-Analysis" OR DE "Psychotherapeutic Counseling" OR DE "Family Therapy" OR DE "Conjoint Therapy" OR DE "Structural Family Therapy" OR DE "Psychotherapeutic Techniques" OR DE "Active Listening" OR DE "Centering" OR DE "Cotherapy" OR DE "Free Association" OR DE "Life Review" OR DE "Mirroring" OR DE "Morita Therapy" OR DE "Motivational Interviewing" OR DE "Mutual Storytelling Technique" OR DE "Paradoxical Techniques" OR DE "Self-Affirmation")		
S3	S1 OR S2	Expanders - Apply equivalent subjects<br>Search modes - Find all my search terms	133,329
S2	TI ((alzheimer* OR amentia* OR "benson* syndrome*" OR "binswanger* disease*" OR "binswanger* encephalopath*" OR dementia* OR "familial pick* disease*" OR "lewy body disease*" OR	Expanders - Apply equivalent subjects<br>Search modes - Find all my search terms	130,460

	"mesulam* syndrome*" OR "posterior cortical atroph*" OR "primary progressive aphasia*" OR "subcortical leukoencephalopath*" OR "wilhelmsen lynch disease*")) OR AB ((alzheimer* OR amentia* OR "benson* syndrome*" OR "binswanger* disease*" OR "binswanger* encephalopath*" OR dementia* OR "familial pick* disease*" OR "lewy body disease*" OR "mesulam* syndrome*" OR "posterior cortical atroph*" OR "primary progressive aphasia*" OR "subcortical leukoencephalopath*" OR "wilhelmsen lynch disease*")) OR KW ((alzheimer* OR amentia* OR "benson* syndrome*" OR "binswanger* disease*" OR "binswanger* encephalopath*" OR dementia* OR "familial pick* disease*" OR "lewy body disease*" OR "mesulam* syndrome*" OR "posterior cortical atroph*" OR "primary progressive aphasia*" OR "subcortical leukoencephalopath*" OR "wilhelmsen lynch disease*"))		
S1	DE "Dementia" OR DE "Alzheimer's Disease" OR DE "Dementia with Lewy Bodies" OR DE "Frontotemporal Lobar Degeneration" OR DE "Vascular Dementia"	Expanders - Apply equivalent subjects<br>Search modes - Find all my search terms	102,012

6. CINAHL

Interface: **EBSCOhost** - content coverage from 1981<br><br>Date of Search: 28 March 2025<br><br>Number of hits: 6,780	Field labels<br><br>• MH+ = exploded CINAHL Heading<br>• MH = non exploded CINAHL Heading<br>• TI = title<br>• AB = abstract<br>• Nx = within x words, regardless of order<br>• * = truncation of word for alternate endings<br>• # = 0-1 letter/number<br>• ? = 1 letter/number<br><br>Note: sometimes "quotation marks" are needed for single search terms to avoid automatic term mapping (lemmatization)

#	Query	Limiters/Expanders	Results
S53	S3 AND S42 AND S50	Limiters - Publication Date: 20150101-20251231<br>Expanders - Apply equivalent subjects<br>Narrow by Language: - english<br>Search modes - Find all my search terms	6,780
S52	S3 AND S42 AND S50	Limiters - Publication Date: 20150101-20251231<br>Expanders - Apply equivalent subjects<br>Search modes - Find all my search terms	6,955
S51	S3 AND S42 AND S50	Expanders - Apply equivalent subjects<br>Search modes - Find all my search terms	11,026
S50	S43 OR S44 OR S45 OR S46 OR S47 OR S48 OR S49	Expanders - Apply equivalent subjects<br>Search modes - Find all my search terms	3,966,879
S49	TI ((acceptability OR adapt* OR adherence OR adoption* OR advantage* OR appropriateness OR barrier# OR "business model*" OR cost# OR create OR customis* OR customiz* OR deliver OR design* OR develop* OR document* OR effect# OR effectiveness OR efficacy OR evaluat* OR	Expanders - Apply equivalent subjects<br>Search modes - Find all my search terms	3,750,049

	exploratory OR facilitator* OR feasibility OR fidelity OR impact* OR implement* OR "mechanism* of change" OR penetration OR pilot OR plan OR produce OR production* OR proof-of-concept OR quality OR refine OR sustainability OR testing OR theory OR theoretical OR theories OR trial# OR trialability OR uncertainty OR uptake* OR usability OR user-centered)) OR AB ((acceptability OR adapt* OR adherence OR adoption* OR advantage* OR appropriateness OR barrier# OR "business model*" OR cost# OR create OR customis* OR customiz* OR deliver OR design* OR develop* OR document* OR effect# OR effectiveness OR efficacy OR evaluat* OR exploratory OR facilitator* OR feasibility OR fidelity OR impact* OR implement* OR "mechanism* of change" OR penetration OR pilot OR plan OR produce OR production* OR proof-of-concept OR quality OR refine OR sustainability OR testing OR theory OR theoretical OR theories OR trial# OR trialability OR uncertainty OR uptake* OR usability OR user-centered))		
S48	(MH "Clinical Trials+")	Expanders - Apply equivalent subjects<br>Search modes - Find all my search terms	361,700
S47	(MH "Costs and Cost Analysis+")	Expanders - Apply equivalent subjects<br>Search modes - Find all my search terms	139,692
S46	(MH "Implementation Science")	Expanders - Apply equivalent subjects<br>Search modes - Find all my search terms	1,753
S45	(MH "Pilot Studies")	Expanders - Apply equivalent subjects<br>Search modes - Find all my search terms	97,552
S44	(MH "Evaluation Research+")	Expanders - Apply equivalent subjects<br>Search modes - Find all my search terms	343,621

S43	(MH "Program Development+")	Expanders - Apply equivalent subjects<br>Search modes - Find all my search terms	112,189
S42	S4 OR S5 OR S6 OR S7 OR S8 OR S9 OR S10 OR S11 OR S12 OR S13 OR S14 OR S15 OR S16 OR S17 OR S18 OR S19 OR S20 OR S21 OR S22 OR S23 OR S24 OR S25 OR S26 OR S27 OR S28 OR S29 OR S30 OR S31 OR S32 OR S33 OR S34 OR S35 OR S36 OR S37 OR S38 OR S39 OR S40 OR S41	Expanders - Apply equivalent subjects<br>Search modes - Find all my search terms	543,754
S41	TI (home* N2 modification*) OR AB (home* N2 modification*)	Expanders - Apply equivalent subjects<br>Search modes - Find all my search terms	579
S40	TI (("built environment*" OR "environment* adaptation*")) OR AB (("built environment*" OR "environment* adaptation*"))	Expanders - Apply equivalent subjects<br>Search modes - Find all my search terms	2,664
S39	TI (((architectural OR environmental* OR facility OR facilities OR garden* OR "human centered" OR residential OR universal) N2 design*)) OR AB (((architectural OR environmental* OR facility OR facilities OR garden* OR "human centered" OR residential OR universal) N2 design*))	Expanders - Apply equivalent subjects<br>Search modes - Find all my search terms	3,288
S38	TI ((("care planning" OR "end of life" OR psychoeducation* OR psycho-education* OR "palliative care") N2 (intervention* OR program* OR therap* OR treatment*))) OR AB ((("care planning" OR "end of life" OR psychoeducation* OR psycho-education* OR "palliative care") N2 (intervention* OR program* OR therap* OR treatment*)))	Expanders - Apply equivalent subjects<br>Search modes - Find all my search terms	7,722
S37	TI (((animal* OR human* OR pet# OR social) N2 robot*)) OR AB (((animal* OR human* OR pet# OR social) N2 robot*))	Expanders - Apply equivalent subjects<br>Search modes - Find all my search terms	869
S36	TI (((assistive OR companion*) N2 (animal# OR pet#))) OR AB (((assistive OR companion*) N2 (animal# OR pet#)))	Expanders - Apply equivalent subjects<br>Search modes - Find all my search terms	584

S35	TI (((animal* OR "animal assisted" OR dog# OR equine* OR pet# OR "pet assisted") N2 ("emotional support" OR intervention* OR therap*))) OR AB (((animal* OR "animal assisted" OR dog# OR equine* OR pet# OR "pet assisted") N2 ("emotional support" OR intervention* OR therap*)))	Expanders - Apply equivalent subjects Search modes - Find all my search terms	2,602
S34	TI ("meeting centre*" N2 program*) OR AB ("meeting centre*" N2 program*)	Expanders - Apply equivalent subjects Search modes - Find all my search terms	15
S33	TI (("social activit*" N2 (intervention* OR program* OR therap* OR treatment*))) OR AB (("social activit*" N2 (intervention* OR program* OR therap* OR treatment*)))	Expanders - Apply equivalent subjects Search modes - Find all my search terms	100
S32	TI (("social interaction intervention*" OR "social intervention*")) OR AB (("social interaction intervention*" OR "social intervention*"))	Expanders - Apply equivalent subjects Search modes - Find all my search terms	812
S31	TI (((alzheimer* OR dementia* OR memory) N2 cafe#)) OR AB (((alzheimer* OR dementia* OR memory) N2 cafe#))	Expanders - Apply equivalent subjects Search modes - Find all my search terms	54
S30	TI ((community N2 (activit* OR hub# OR initiative* OR intervention* OR program*))) OR AB ((community N2 (activit* OR hub# OR initiative* OR intervention* OR program*)))	Expanders - Apply equivalent subjects Search modes - Find all my search terms	23,711
S29	TI ((museum* N2 (activit* OR attend* OR alzheimer* OR dementia* OR intervention* OR participat* OR program* OR therap* OR visit))) OR AB ((museum* N2 (activit* OR attend* OR alzheimer* OR dementia* OR intervention* OR participat* OR program* OR therap* OR visit)))	Expanders - Apply equivalent subjects Search modes - Find all my search terms	124
S28	TI ((behavio* W1 ("change technique*" OR counsel* OR modification* OR therap* OR treatment* OR support))) OR AB ((behavio* W1 ("change technique*" OR counsel* OR modification* OR therap* OR treatment* OR support)))	Expanders - Apply equivalent subjects Search modes - Find all my search terms	24,781
S27	TI (((art OR choir* OR music OR writing) N2 (activit* OR alzheimer* OR dementia* OR making OR intervention* OR participat* OR program* OR	Expanders - Apply equivalent subjects	18,406

	therap* OR treatment*))) OR AB (((art OR choir* OR music OR writing) N2 (activit* OR alzheimer* OR dementia* OR making OR intervention* OR participat* OR program* OR therap* OR treatment*)))	Search modes - Find all my search terms	
S26	TI ((("health educat*" OR nonpharmacolog* OR non-pharmacolog*) N2 (intervention* OR program* OR therap* OR treatment*))) OR AB ((("health educat*" OR nonpharmacolog* OR non-pharmacolog*) N2 (intervention* OR program* OR therap* OR treatment*)))	Expanders - Apply equivalent subjects Search modes - Find all my search terms	11,559
S25	TI (((cognition OR cognitive OR "cognitive behavio*") N1 (intervention* OR program* OR rehabilitation OR reframing OR remediation OR restructuring OR therap* OR training))) OR AB (((cognition OR cognitive OR "cognitive behavio*") N1 (intervention* OR program* OR rehabilitation OR reframing OR remediation OR restructuring OR therap* OR training)))	Expanders - Apply equivalent subjects Search modes - Find all my search terms	23,486
S24	TI (((handicraft* OR poetry OR reading OR singing OR storytelling OR theatre#) N2 (activit* OR intervention* OR program* OR therap* OR treatment*))) OR AB (((handicraft* OR poetry OR reading OR singing OR storytelling OR theatre#) N2 (activit* OR intervention* OR program* OR therap* OR treatment*)))	Expanders - Apply equivalent subjects Search modes - Find all my search terms	2,284
S23	TI (("acoustic stimulation" OR "anger management" OR "applied behavio#r analys?s" OR aroma OR "auditory stimulation" OR choir* OR "communication training" OR "conversation* coaching" OR "compensatory strateg*" OR clown# OR "creative writing" OR doll# OR drama OR "emotion* focused" OR "encounter group*" OR "forest bath*" OR "free association*" OR garden* OR "grief counseling" OR "guided imagery" OR horticultur* OR laughter OR handicraft* OR laying-on-of-hands OR "life story work" OR massage OR meditation OR mindfulness OR "mixed realit*" OR "multisensory stimulation" OR museum* OR music OR "namaste care" OR "peer group*" OR "personal validation" OR photo-voice OR poetry OR psychodrama OR reablement OR "reality orientation" OR reiki OR "relaxation techni*" OR reminiscence OR "role playing*" OR sandplay OR "sensory stimulation" OR shinrin-yoku OR snoezelen OR	Expanders - Apply equivalent subjects Search modes - Find all my search terms	39,357

	singing OR storytelling OR "systematic therap*" OR t-group* OR theatre#))		
S22	TI ((("acceptance and commitment" OR "acoustic stimulation" OR "anger management" OR "applied behavio#r analys?s" OR aroma OR aversion OR "auditory stimulation" OR aversive OR behavior-change OR "client centered" OR clown OR colo#r OR compassion* OR "communication training" OR "compensatory strateg*" OR conditioning OR "conversation* coaching" OR coping OR culture-based OR couple OR "dialectical behavio#r*" OR dignity OR "directed reverie" OR doll# OR drama OR "emotion* focused" OR "encounter group*" OR forest OR "free association*" OR garden* OR gestalt OR grief OR "guided imagery" OR "health educat*" OR healing OR horticultur* OR laughter OR "life review" OR laying-on-of-hands OR "life story work" OR marital OR marriage OR massage OR meditation OR mindfulness OR "mixed realit*" OR "multi family" OR multifamily OR "multiple family" OR multisensory OR multi-sensory OR "namaste care" OR nature OR nondirective OR "object handling" OR "peer group*" OR "personal validation" OR photo-voice OR "play-based mental health" OR psychoanalytic* OR psychodrama OR psycholog* OR psychosocial* OR psycho-social* OR reablement OR reality OR reflex OR reiki OR relaxation OR reminiscence OR rogerian OR "role playing*" OR sandplay OR "sensory stimulation" OR shinrin-yoku OR snoezelen OR "social support" OR socioenvironment* OR socio-environment* OR "solution focused brief" OR t-group* OR talking OR touch*) N2 (intervention* OR program* OR therap* OR treatment*))) OR AB ((("acceptance and commitment" OR "acoustic stimulation" OR "anger management" OR "applied behavio#r analys?s" OR aroma OR aversion OR "auditory stimulation" OR aversive OR behavior-change OR "client centered" OR clown OR colo#r OR compassion* OR "communication training" OR "compensatory strateg*" OR conditioning OR "conversation* coaching" OR coping OR culture-based OR couple OR "dialectical behavio#r*" OR dignity OR "directed reverie" OR doll# OR drama OR "emotion* focused" OR "encounter group*" OR forest OR "free association*" OR garden* OR gestalt OR grief OR "guided imagery" OR "health educat*" OR healing OR horticultur* OR laughter OR "life review" OR laying-on-of-hands OR "life story work" OR marital	Expanders - Apply equivalent subjects<br>Search modes - Find all my search terms	59,908

	OR marriage OR massage OR meditation OR mindfulness OR "mixed realit*" OR "multi family" OR multifamily OR "multiple family" OR multisensory OR multi-sensory OR "namaste care" OR nature OR nondirective OR "object handling" OR "peer group*" OR "personal validation" OR photo-voice OR "play-based mental health" OR psychoanalytic* OR psychodrama OR psycholog* OR psychosocial* OR psycho-social* OR reablement OR reality OR reflex OR reiki OR relaxation OR reminiscence OR rogerian OR "role playing*" OR sandplay OR "sensory stimulation" OR shinrin-yoku OR snoezelen OR "social support" OR socioenvironment* OR socio-environment* OR "solution focused brief" OR t-group* OR talking OR touch*) N2 (intervention* OR program* OR therap* OR treatment*)))		
S21	TI (("crisis intervention*" OR "self-help group*" OR "sensitivity training group*")) OR AB (("crisis intervention*" OR "self-help group*" OR "sensitivity training group*"))	Expanders - Apply equivalent subjects Search modes - Find all my search terms	1,861
S20	TI (((caregiver OR complementary OR family OR group OR narrative OR occupational OR play) W1 (intervention* OR program* OR therap* OR treatment*))) OR AB (((caregiver OR complementary OR family OR group OR narrative OR occupational OR play) W1 (intervention* OR program* OR therap* OR treatment*)))	Expanders - Apply equivalent subjects Search modes - Find all my search terms	68,575
S19	TI ((aromatherap* OR bibliotherap* OR chromatotherap* OR chromotherap* OR dramatherap* OR ecotherap* OR psychotherap* OR reflexotherap*)) OR AB ((aromatherap* OR bibliotherap* OR chromatotherap* OR chromotherap* OR dramatherap* OR ecotherap* OR psychotherap* OR reflexotherap*))	Expanders - Apply equivalent subjects Search modes - Find all my search terms	20,806
S18	(MH "Facility Design and Construction+")	Expanders - Apply equivalent subjects Search modes - Find all my search terms	16,658
S17	(MH "Built Environment") OR (MH "Home Environment") OR (MH "Environment, Controlled+")	Expanders - Apply equivalent subjects Search modes - Find all my search terms	30,658

S16	(MH "Palliative Care")	Expanders - Apply equivalent subjects<br>Search modes - Find all my search terms	44,851
S15	(MH "Advance Care Planning")	Expanders - Apply equivalent subjects<br>Search modes - Find all my search terms	5,594
S14	(MH "Support, Social+")	Expanders - Apply equivalent subjects<br>Search modes - Find all my search terms	116,236
S13	(MH "Support Groups")	Expanders - Apply equivalent subjects<br>Search modes - Find all my search terms	11,894
S12	(MH "Singing")	Expanders - Apply equivalent subjects<br>Search modes - Find all my search terms	3,949
S11	(MH "Therapy Animals")	Expanders - Apply equivalent subjects<br>Search modes - Find all my search terms	85
S10	(MH "Occupational Therapy")	Expanders - Apply equivalent subjects<br>Search modes - Find all my search terms	23,805
S9	(MH "Creative Arts Therapy+")	Expanders - Apply equivalent subjects<br>Search modes - Find all my search terms	12,837
S8	(MH "Massage")	Expanders - Apply equivalent subjects<br>Search modes - Find all my search terms	15,457
S7	(MH "Therapeutic Touch")	Expanders - Apply equivalent subjects	1,612

		Search modes - Find all my search terms	
S6	(MH "Mental Healing")	Expanders - Apply equivalent subjects<br>Search modes - Find all my search terms	1,253
S5	(MH "Aromatherapy")	Expanders - Apply equivalent subjects<br>Search modes - Find all my search terms	2,947
S4	(MH "Psychotherapy+")	Expanders - Apply equivalent subjects<br>Search modes - Find all my search terms	248,635
S3	S1 OR S2	Expanders - Apply equivalent subjects<br>Search modes - Find all my search terms	116,458
S2	TI ((alzheimer* OR amentia* OR "benson* syndrome*" OR "binswanger* disease*" OR "binswanger* encephalopath*" OR dementia* OR "familial pick* disease*" OR "lewy body disease*" OR "mesulam* syndrome*" OR "posterior cortical atroph*" OR "primary progressive aphasia*" OR "subcortical leukoencephalopath*" OR "wilhelmsen lynch disease*")) OR AB ((alzheimer* OR amentia* OR "benson* syndrome*" OR "binswanger* disease*" OR "binswanger* encephalopath*" OR dementia* OR "familial pick* disease*" OR "lewy body disease*" OR "mesulam* syndrome*" OR "posterior cortical atroph*" OR "primary progressive aphasia*" OR "subcortical leukoencephalopath*" OR "wilhelmsen lynch disease*"))	Expanders - Apply equivalent subjects<br>Search modes - Find all my search terms	98,296
S1	(MH "Dementia") OR (MH "Alzheimer's Disease") OR (MH "Dementia, Vascular") OR (MH "Frontotemporal Lobar Degeneration") OR (MH "Frontotemporal Dementia") OR (MH "Lewy Body Disease") OR (MH "Mixed Dementias")	Expanders - Apply equivalent subjects<br>Search modes - Find all my search terms	83,871
