# Supplementary material for: Methodologies and methods for the development, evaluation and implementation of psychosocial interventions for dementia: protocol for a scoping review
Source: BMJ Open. 2026 Apr 24;16(4):e114584. doi: 10.1136/bmjopen-2025-114584 (PMC13110686; doi:10.1136/bmjopen-2025-114584)
Supplement: Supplementary file 4 [file bmjopen-2025-114584.reviewer_comments.pdf]

PEER REVIEW HISTORY

BMJ Open publishes all reviews undertaken for accepted manuscripts. Reviewers are asked to complete a checklist review form and are provided with free text boxes to elaborate on their assessment. These free text comments are reproduced below.

ARTICLE DETAILS

Title (Provisional)

Methodologies and methods for the development, evaluation, and implementation of psychosocial interventions for dementia: Protocol for a scoping review

Authors

D'andrea, Federica; Bartels, Sara Laureen; Markaryan, Marine; Masterson-Algar, Patricia; Bernal, Andrea N; De Bruin, Simone R; Chirico, Ilaria; Flynn, Aisling; Garcia, Lesley; Gebhard, Doris; Handley, Melanie; Janssen, Niels; Roes, Martina; Stephens, Nathan; Teesing, Gwen; Van den Block , Lieve; Windle, Karen; Moniz-Cook , Esme; Graff, Maud

---

VERSION 1 - REVIEW

Reviewer 1

Name Hartigan, Irene

Affiliation University College Cork National University of Ireland, Catherine McAuley School of Nursing and Midwifery

Date 22-Dec-2025

COI None

Thank you for making revisions to the original version of this manuscript

• Several sections of the manuscript exhibit repetitive phrasing, duplicated content blocks (e.g., Abstract, Methods, tables, and figures reappearing across page ranges), and inconsistent terminology/notation (e.g., “debilitating” vs “depilating,” decimal separators in p-values). See pdf attached with some sections highlighted with these errors. These features reduce clarity and may reflect automated paraphrasing, translation, or repeated export/merge during manuscript preparation.

• To improve readability and scientific rigor, please (i) remove duplicated sections and standardize statistical notation; (ii) correct grammatical and lexical errors (e.g., “six out of eight,” “debilitating”); (iii) ensure each claim is anchored to explicit in-text citations; and (iv) provide a concise, non-redundant Methods description with full, reproducible search strings and a single PRISMA flow diagram.

• Ampersand in consistently used thought the manuscript. If any AI-assisted tools (e.g., language models, paraphrasing or translation software) were used during drafting, please disclose this in line with journal policy (e.g., under ‘Acknowledgments’ or ‘Author contributions’) and confirm that all scientific interpretation, data extraction, and bias assessment were performed and verified by the authors.

• Variation exists in p value notation (p = 0.003 vs p = 0,003), confidence interval style, and formatting (punctuation). Please round up or down percentages.

• This paper is more acceptable for an exploratory or scoping review, for a systematic review aiming to inform practice, the scope should ideally be narrower or stratified: (1) Separate analyses for paediatric vs. adult patients (2) Consider subgrouping by cancer type or treatment phase.

• The inclusion of cancer and leukaemia patients who are among the most immunocompromised populations, in interventions involving direct contact with dogs raises significant ethical and clinical concerns. While animal-assisted therapy may offer psychological benefits, the manuscript does not provide sufficient justification for exposing such vulnerable patients to potential microbial risks. Infection control measures are mentioned only briefly (e.g., prohibition of licking in one study), but there is no comprehensive description of protocols across included studies.

• Given the documented presence of pathogens in therapy animals and the high susceptibility of oncology patients to infections, the authors should clearly outline the infection prevention strategies employed in the reviewed studies (e.g., certification of therapy dogs, hygiene protocols, screening for zoonotic pathogens) and discuss the implications for patient safety. Without this information, the feasibility and ethical appropriateness of these interventions remain questionable.

• The focus of this paper is investigation of the effectiveness of dog – assisted therapy on the emotional well – being of cancer or leukaemia patients, both children and adults. Eight studies is a very small evidence base for a systematic review, especially when split across two distinct populations. This limits the ability to generalize findings. With only 3 adult studies, of which 2 were related to patients and one to staff, and 5 paediatric studies, subgroup analysis is not robust. Any conclusions about populations-specific effects would be tentative at best.

• Six out of eight included studies used convenience sampling, which limits representativeness and generalizability of findings. This should be discussed more critically in the limitations section, emphasizing how convenience sampling may introduce selection bias and affect external validity.

• Keywords are not reflective of the scope of this paper: Animal Assisted – Therapy, Dog Assisted – Therapy, Neoplasms, Emotional Well-Being, Psychological, Quality of Life.

• Page 4 -The opening line is weak and could be restructured for better readability.

• Line of 2nd opening paragraph has the references cited differently to others.

• The population scope is indeed broad covering both adults and children with any type of cancer or leukaemia. This leads to my concerns re: (1) Heterogeneity and (2) Interpretation risk.

• Regarding heterogeneity, there are different cancer types (solid tumours vs. hematologic malignancies) and treatment contexts (chemotherapy, surgery, palliative care, in patient or outpatient clinic) which may influence emotional distress and response to dog assisted therapy. Also, combining paediatric and adult populations introduces variability in psychological needs and intervention delivery.

• There is an interpretation risk as broad inclusion may result in overgeneralization of findings, and without subgroup analysis, the conclusions may lack clinical applicability.

• While the manuscript lists keywords and databases, the search strategy lacks full reproducibility details (e.g., Boolean operators, date ranges, filters applied). For transparency and adherence to PRISMA guidelines, the authors should provide the complete search strings for each database in an appendix or supplementary material.

• Page 5 the search strategy was not optimized and does not seem to be adapted it for other databases. Consider the limited focus also of the outcome-related concepts (psychological, psychosocial, well-being, quality of life is limited). Consider Psychological Well-Being"[MeSH] OR "Stress, Psychological"[MeSH] OR emotional[tiab] OR "emotional well-being"[tiab] OR psychological[tiab] OR psychosocial[tiab] OR "mental health"[tiab]

• This is particularly important (page 6 line 7) exclusion criteria was studies that did not refer to emotional well-being. Please explain.

• The manuscript mentions inclusion of grey literature to reduce bias; however, the approach appears limited to Google Scholar, which is not considered a systematic or reproducible source. This raises concerns about the completeness of the search and potential selection bias. Additionally, grey literature often lacks rigorous peer review, which may affect the reliability of findings. The manuscript does not specify whether grey literature was appraised for methodological quality. Given the potential variability in rigor, the authors should clarify whether quality assessment was applied and, if not, acknowledge this as a limitation.

• It has not been stated the implications to the findings given the wide variation in study designs (RCT, quasi-experimental, prospective), populations (children vs adults), and outcome measures.

• Some studies combined dog-assisted therapy with other interventions (e.g., Certified Child Life Specialist), making it hard to isolate the effect of dogs alone.

• Clarify Intervention Details: Separate effects of dog-assisted therapy from combined interventions.

• Selective Reporting: Conflicting findings for outcomes like cortisol and depression are noted but not explored in depth.

• Overgeneralization: Conclusions emphasize positive effects despite mixed or non-significant results for several indicators.

• Protocol Adherence: PROSPERO registration is mentioned, but deviations from the protocol are not discussed.

• What order are the studies presented in for Table 1?

• Robins 1 table not labelled correctly.

While revisions have been attempted and some improvements are evident compared to the previous version, this manuscript is not currently acceptable for publication. Several critical issues remain unresolved, including consistency in terminology, and methodological transparency. Additionally, concerns about communication protocols and alignment with stated aims persist. These gaps significantly impact the rigor and interpretability of the work.

**Reviewer**	**2**
**Name**	**Bacsu, Juanita-Dawne**
**Affiliation**	**Thompson Rivers University, School of Nursing**
**Date**	**03-Jan-2026**
**COI**	**None**

January 2, 2025

Dear BMJ Open Editorial Office,

Thank you for the opportunity to review the protocol entitled, “Methodologies and methods for the development, evaluation, and implementation of psychosocial interventions for dementia: Protocol for a scoping review.” This scoping review aims to examine the methodological trends, use, and gaps in the development, evaluation, and implementation of psychosocial interventions for dementia, and determine if and how the core elements of the Medical Research Council (MRC) framework were applied. I have included my edits and suggestions below, and I hope that the authors find them helpful.

1. References are not typically included in the abstract, consider rephrasing to delete the citation (p. 6 of 77, line 60).

2. The abstract would be strengthened by having a bit more focus on the rationale, and implications for conducting this scoping review.

3. The strengths and limitations section would be enhanced with some refinement to tighten the points more succinctly.

4. In the strengths and limitations section, bullet #4 requires edits for clarity. Also, in this bullet the word "article" requires an s- Focus is on methodologies and methods used and not on outcomes of interventions and data synthesis is limited to article published in English

5. Throughout the paper, please work to reduce the number of direct quotations and work more on paraphrasing in your own words to make the argument clearer and more cohesive (e.g., page 9 of 77, lines 111-112 and page 10 of 77, lines 139-141).

6. On page 14 of 77, line 12 with the software (e.g., Covidence and ASReview) requires references for the software rather than URL links that are provided in the text below.

7. It would be helpful for the authors to discuss the rationale of the timeframe from 2015 onwards.

8. In the text, it would be useful to discuss whether the Population, Concept, Context (PCC), was followed that is recommended for scoping reviews. See: Peters MDJ, Marnie C, Tricco AC, Pollock D, Munn Z, Alexander L, McInerney P, Godfrey CM, Khalil H. Chapter 11: Scoping Reviews. In: Aromataris E, Munn Z (Editors). JBI Manual for Evidence Synthesis, JBI, 2020

9. In the search strategy, please ensure adequate details are provided to facilitate reproducibility of all study related procedures and analysis. In the text, it would also be helpful to provide an example of a search string for one of the databases that will be used.

10. On page 17 of 77,lines 296-298 (data synthesis section) the tense looks incorrect as it is written in past tense, unless the scoping review has already been completed. Was the scoping review completed in January 2025 or only the pilot screening exercise?

11. On page 17 of 77, lines 298-301, it would be useful to discuss how thematic analysis will be conducted to categorize the themes. For example, will the authors be using a thematic analysis framework, will inductive or deductive thematic analysis be used, will thematic maps or theme piles be used, and how will the themes be created within each of the varying categories. Software (EviAtlas) is briefly mentioned but few other details are provided and the software doesn't create the themes.

12. Overall, the article would be strengthened with a clearer rationale for the need for the study as well as the implications. This could be bolstered in the abstract, introduction, and conclusion sections.

13. Linked to the above comment, the actual objective needs to be clarified. For example, why is it important to know what the methodological trends, and gaps in the development, evaluation, and implementation of psychosocial interventions for dementia, and how the core elements of the Medical Research Council (MRC) framework were applied.

14. On page 20 of 77, lines 347-350, it is mentioned that people with lived experience will participate in co-designing (lines 349-350). It would be helpful to know how the people with lived experience will contribute to the search strategy, analysis, or interpretation of the results?

15. Also, because this study involves co-design with people who have lived experience of dementia, please clarify what ethical approval process was followed. This ethical process needs to be addressed on page 18 of 77 under the ethics section.

16. In the knowledge dissemination section (page 18 of 77, 313-14), it would be helpful to provide more information about the dissemination. For example, are you targeting your dissemination to specific audiences and through which mediums? More specifically, what strategies will be used to target knowledge dissemination to different groups?

Reviewer **3**

Name **Laver, Kate**

Affiliation **Flinders University, Flinders University, Department of Rehabilitation**

Date **07-Jan-2026**

COI **None**

This is a very thorough protocol with sound methods. The use of AI is novel but well explained and justified. I look forward to reading the results of this review when complete.

My only comment is that the tense seems to change at times throughout the protocol and need to ensure (given its a protocol) that everything is in the same tense

---

VERSION 1 - AUTHOR RESPONSE

Reviewer: 2
Dr. Juanita-Dawne Bacsu, Thompson Rivers University
Comments to the Author:

January 2, 2025
Dear BMJ Open Editorial Office,
Thank you for the opportunity to review the protocol entitled, “Methodologies and methods for the development, evaluation, and implementation of psychosocial interventions for dementia: Protocol for a scoping review.” This scoping review aims to examine the methodological trends, use, and gaps in the development, evaluation, and implementation of psychosocial interventions for dementia, and determine if and how the core elements of the Medical Research Council (MRC) framework were applied. I have included my edits and suggestions below, and I hope that the authors find them helpful.
1. References are not typically included in the abstract, consider rephrasing to delete the citation (p. 6 of 77, line 60).

>>>> We thank the reviewer for this comment and agree that references are not typically included in the abstract. We have therefore removed the citation from the abstract (Page 4, line 64).

2. The abstract would be strengthened by having a bit more focus on the rationale, and implications for conducting this scoping review.

>>>>Thank you for this helpful feedback. We have clarified the rationale in the abstract background (Page 4, lines 57–61) and have expanded on the implications in the "Ethics and Dissemination" section (Page 5, lines 81–87).

3. The strengths and limitations section would be enhanced with some refinement to tighten the points more succinctly.

>>> Thank you for this comment. We have refined the Strengths and Limitations section to make the points clearer and more concise (Page 6, lines 98-112). In response to the Editor's comment, we have removed Point 1 (Page 6, lines 95-97), as it was focused on the novelty of the study rather than on the methods.

4. In the strengths and limitations section, bullet #4 requires edits for clarity. Also, in this bullet the word "article" requires an s- Focus is on methodologies and methods used and not on outcomes of interventions and data synthesis is limited to article published in English

>>>> To enhance clarity, we have split this statement into two separate points and revised the wording (Page 6, lines 106-109).

5. Throughout the paper, please work to reduce the number of direct quotations and work more on paraphrasing in your own words to make the argument clearer and more cohesive (e.g., page 9 of 77, lines 111-112 and page 10 of 77, lines 139-141).

>>>>We have removed the direct quote from Skivington et al. (2024) on page 7, lines 122–123 and rephrased it in our own words (Page 7, lines 125-127). The quote on page 9, lines 156–159 has also been removed and appropriately paraphrased in Page 8, lines 152-156; Page 9, lines 164-168; and Page 10, lines 187-190.

6. On page 14 of 77, line 12 with the software (e.g., Covidence and ASReview) requires references for the software rather than URL links that are provided in the text below.

>>>>We have now added a reference for Covidence (Page 14, line 269; line 286) and ASReview on Page 14, line 273, replacing the previous URL links.

7. It would be helpful for the authors to discuss the rationale of the timeframe from 2015 onwards.

>>>>We thank the reviewer for this helpful comment. We have added a sentence in the Methods section (page 12, line 241-242) explaining that the timeframe from 2015 onwards was chosen because this corresponds to the publication of MRC process evaluation guidance relevant to our research questions. Earlier MRC guidance focused on randomised trials and did not address process evaluation.

8. In the text, it would be useful to discuss whether the Population, Concept, Context (PCC), was followed that is recommended for scoping reviews. See: Peters MDJ, Marnie C, Tricco AC, Pollock D, Munn Z, Alexander L, McInerney P, Godfrey CM, Khalil H. Chapter 11: Scoping Reviews. In: Aromataris E, Munn Z (Editors). JBI Manual for Evidence Synthesis, JBI, 2020

>>>> In this review, the Population, Concept and Context (PCC) framework guided the development of the protocol. Population, concept and context were clearly defined and informed the identification of key terms, the development of the inclusion criteria, and the data extraction process. This has now been clearly reported in the Methods section (page 13, lines 253-254).

9. In the search strategy, please ensure adequate details are provided to facilitate reproducibility of all study related procedures and analysis. In the text, it would also be helpful to provide an example of a search string for one of the databases that will be used.
>>>>We agree with the reviewer on the importance of reproducibility of the study procedure. Accordingly, we have included again the full search strategy in the resubmission, with search strings for each database provided in the supplementary materials.

10. On page 17 of 77,lines 296-298 (data synthesis section) the tense looks incorrect as it is written in past tense, unless the scoping review has already been completed. Was the scoping review completed in January 2025 or only the pilot screening exercise?

>>>> We thank the reviewer for highlighting the typo. The use of past tense in the Data Synthesis section (Page 17, lines 339–341) was misleading. Only the pilot test of the screening process was completed in January 2025. The section has now been revised with the use of future tense. Based on the third reviewer's comment, we have now presented the 'Methods and analysis' heading section outlining the review procedure in the future tense.

11. On page 17 of 77, lines 298-301, it would be useful to discuss how thematic analysis

will be conducted to categorize the themes. For example, will the authors be using a thematic analysis framework, will inductive or deductive thematic analysis be used, will thematic maps or theme piles be used, and how will the themes be created within each of the varying categories. Software (EviAtlas) is briefly mentioned but few other details are provided and the software doesn't create the themes.

>>>>We thank the reviewer for this comment. We would like to clarify that our scoping review does not use formal thematic analysis. Studies will be categorized according to the review objectives (e.g., methodologies, methods, MRC framework phases and whether the study address the six core elements). The results and potential trends and gaps will be summarized using percentages and visualizations (e.g., tables, charts, histograms). EviAtlas will support clear visualizations but does not generate themes. The manuscript has been revised to clarify this and remove wording that could suggest a formal thematic analysis (Page 17, lines 341-351).

12. Overall, the article would be strengthened with a clearer rationale for the need for the study as well as the implications. This could be bolstered in the abstract, introduction, and conclusion sections.

We thank the reviewer for this valuable suggestion. We agree that it is useful to further elaborate on the rationale for the need for this study and to more explicitly articulate its implications. Accordingly, we have revised the abstract (Page 4, lines 57-61), introduction (Pages 8-9, lines 152-156; line 164-169; Pages 9-10, lines 174-191) and dissemination (Pages 17-18, lines 356-364). These revisions emphasise the importance of research methods and methodology in the development, evaluation and implementation of relevant, effective feasible and scalable interventions. While the MRC framework provides overarching guidance for advancing complex intervention research, there remains limited practical guidance for dementia researchers on how its core elements can be addressed within predefined project timelines and funding constraints.

To date, despite the rapid growth of psychosocial dementia research, to the authors' knowledge there is currently no evidence synthesis summarising research methods and methodologies used within this field. By synthesising the methods and methodologies applied, this study provides an overview of current approaches, identifies patterns (such as methods/methodologies used across time and geographical contexts), and highlights underdeveloped phases, methodological weaknesses, and practical challenges, as well as examples of good practice. This work therefore contributes to advancing psychosocial dementia research and, in turn, helps reduce the gap between research and practice.

13. Linked to the above comment, the actual objective needs to be clarified. For

example, why is it important to know what the methodological trends, and gaps in the development, evaluation, and implementation of psychosocial interventions for dementia, and how the core elements of the Medical Research Council (MRC) framework were applied.

>>>>We have clarified the rationale beyond the objectives of interest, as outlined in our response to reviewer's comment number 12. Understanding the methods and methodologies used in psychosocial dementia research is important because it allows us to map current practices, identify trends, and assess the extent to which the six MRC core elements for advancing complex intervention research have been implemented over the past 10 years. This approach also helps to highlight changes in methods and methodologies, gaps, such as phases of intervention research that are neglected. Such knowledge can inform better research practices, guide future iterations of the MRC framework, and ultimately enhance the quality, relevance, and real-world applicability of psychosocial interventions for people living with dementia.

14. On page 20 of 77, lines 347-350, it is mentioned that people with lived experience will participate in co-designing (lines 349-350). It would be helpful to know how the people with lived experience will contribute to the search strategy, analysis, or interpretation of the results?

>>>> We thank the reviewer for this comment. We would like to clarify that the section referenced (Page 20, lines 418–422) describes the involvement of people living with dementia in the planned Delphi study, rather than the scoping review itself. Both the scoping review and the Delphi study are part of the METHODEM project. The results of the scoping review will inform the Delphi study, which will involve people with lived experience of dementia in co-designing aspects such as item development and interpretation of findings. As this might be potentially confusing, we removed it from the manuscript and adjusted the Patient and Public Involvement statement accordingly.

15. Also, because this study involves co-design with people who have lived experience of dementia, please clarify what ethical approval process was followed. This ethical process needs to be addressed on page 18 of 77 under the ethics section.

>>>>We thank the reviewer for raising this point. As mentioned above, we clarify that the scoping review itself did not involve people living with dementia, and therefore no ethical approval was required for this study. However, in the manuscript (page 19, lines 389-390) we have now included information about the ethical approvals now sought for the Delphi study mentioned, which will involve people with lived experience of dementia, from both Maastricht University (FHML-REC/2025/078) and the University of West London (UWL/REC/SBS-01195).

16. In the knowledge dissemination section (page 18 of 77, 313-14), it would be helpful to provide more information about the dissemination. For example, are you targeting your dissemination to specific audiences and through which mediums? More specifically, what strategies will be used to target knowledge dissemination to different groups?

>>>>We thank the reviewer for this helpful comment. We have expanded the dissemination section to provide more detail on the target audiences and dissemination strategies (Page 18, lines 365-372). Overall, the study will adopt a multi-modal dissemination approach, targeting dementia researchers, methodologists, funding bodies, policymakers, dementia charities, clinicians, people living with dementia, and carers. Results will be shared through peer-reviewed publications, seminars, webinars, education at relevant master programs at universities and conference presentations. Wider dissemination will include blogs, commissioner funder briefing lay summaries on social media, and presentations at local community events involving people living with dementia and carers.

Reviewer: 3
Dr. Kate Laver, Flinders University
Comments to the Author:

This is a very thorough protocol with sound methods. The use of AI is novel but well explained and justified. I look forward to reading the results of this review when complete.
My only comment is that the tense seems to change at times throughout the protocol and need to ensure (given its a protocol) that everything is in the same tense.

>>>>We thank the reviewer for this helpful observation. We have carefully reviewed the manuscript and revised it to ensure consistent use of tense throughout. In line with standard protocol reporting, all sections describing planned methods and procedures are now presented in the future tense.

Moreover, we would like to inform the editor and reviewers that we made one additional adaptation to the protocol and study data extraction. In the resubmission, the rational for and limitations/ benefits of methodological approaches were removed from the manuscript (Page 16, Line 328-329) as they will not be extracted per study but rather included in the discussion exemplary, to streamline the data extraction process.
